# Glial cells maintain synapses by inhibiting an activity-dependent retrograde protease signal

**DOI:** 10.1371/journal.pgen.1007948

**Published:** 2019-03-14

**Authors:** Thomas W. Gould, Bertha Dominguez, Fred de Winter, Gene W. Yeo, Patrick Liu, Balaji Sundararaman, Thomas Stark, Anthony Vu, Jay L. Degen, Weichun Lin, Kuo-Fen Lee

**Affiliations:** 1 Peptide Biology Laboratories, Salk Institute, La Jolla, CA, United States of America; 2 Department of Physiology and Cell Biology, University of Nevada School of Medicine, Reno, NV, United States of America; 3 Department of Neuroregeneration, Netherlands Institute for Neuroscience, Amsterdam, Netherlands; 4 Department of Cellular and Molecular Medicine, Sanford Consortium for Regenerative Medicine, Stem Cell Program and Institute for Genomic Medicine, University of California at San Diego, La Jolla, CA, United States of America; 5 Division of Experimental Hematology, Cancer and Blood Diseases Institute, Cincinnati Children’s Hospital Research Foundation, Cincinnati, OH, United States of America; 6 Department of Neuroscience, University of Texas Southwestern Medical Center, Dallas, TX, United States of America; The Jackson Laboratory, UNITED STATES

## Abstract

Glial cells regulate multiple aspects of synaptogenesis. In the absence of Schwann cells, a peripheral glial cell, motor neurons initially innervate muscle but then degenerate. Here, using a genetic approach, we show that neural activity-regulated negative factors produced by muscle drive neurodegeneration in Schwann cell-deficient mice. We find that thrombin, the hepatic serine protease central to the hemostatic coagulation cascade, is one such negative factor. Trancriptomic analysis shows that expression of the antithrombins serpin C1 and D1 is significantly reduced in Schwann cell-deficient mice. In the absence of peripheral neuromuscular activity, neurodegeneration is completely blocked, and expression of prothrombin in muscle is markedly reduced. In the absence of muscle-derived prothrombin, neurodegeneration is also markedly reduced. Together, these results suggest that Schwann cells regulate NMJs by opposing the effects of activity-regulated, muscle-derived negative factors and provide the first genetic evidence that thrombin plays a central role outside of the coagulation system.

## Author summary

We utilized genetic methods to examine how Schwann cells prevent degeneration of motor neurons (MNs) in the spinal cord. Blocking peripheral, neuromuscular activity completely rescued MNs and neuromuscular junctions (NMJs) in *erbB3* mutant mice lacking Schwann cells, which normally exhibit profound neurodegeneration. We searched for the molecular basis of this effect by examining the transcriptomes (all of the expressed genes) in the muscle of control mice with Schwann cells and *erbB3* mutant mice without them. We found evidence that a negative signal expressed by muscle was regulated by neural activity and normally blocked by factors produced in Schwann cells. When we eliminated this activity-induced negative signal (thrombin) from muscle, MNs and NMJs were protected in *erbB3* mutants, similar to the effects of eliminating activity. Together, these results suggest that Schwann cells prevent neurodegeneration by inhibiting the effect of activity-induced, muscle-derived negative factors, rather than by providing trophic positive factors.

## Introduction

Synapses are the key elements of neural circuits underlying nervous system function. Therefore, understanding cellular and molecular mechanisms of synapse formation and maintenance is fundamental to neurobiology. During development, establishment of the mature pattern of neuronal connectivity is achieved by the formation, differentiation, refinement and maintenance of specialized synaptic contacts between pre-, peri- and post-synaptic cells. The vertebrate neuromuscular junction (NMJ), a synapse between pre-synaptic motor axons, peri-synaptic Schwann and postsynaptic skeletal muscle cells, has been an excellent model to understand synapse formation and maintenance [1, 2]. Many of the neuronal and muscle-derived factors regulating the spatial and temporal sequence of synaptogenesis have been identified. For example, the muscle-specific kinase (MuSK), low-density lipoprotein receptor-related protein 4 (Lrp4) and adaptor protein rapsyn are required for the formation of the spatially restricted pattern of the postsynaptic apparatus, including acetylcholine receptor (AChR) clusters, known as the endplate band, whereas motor nerve-derived signals such as agrin and acetylcholine (ACh) play opposing roles in the refinement and maintenance of the postsynaptic apparatus [3–13]. Similarly, muscle-derived factors such as β-catenin, Lrp4, fibroblast growth factors and laminin β2 are required for branch positioning, presynaptic differentiation and maturation of motor neurons [14–19].

The molecular signals from Schwann cells that regulate synapse formation and maintenance, however, are presently unknown. One hint comes from studies of mutant mice lacking Schwann cells as a result of the targeted deletion of neuregulin 1 (NRG1) or its erbB2 or erbB3 receptors [20–25]. Despite exhibiting a profound defasciculation, motor axons in Schwann cell-deficient mice accurately navigate to their muscle targets, and, like in wild-type (WT) embryos, nerve terminals make contacts in the middle of muscle fibers at embryonic day 14 (E14) [22]. However, nerve terminals and axons concurrently and completely degenerate by E15.5- E16.5 [22] in a process that we refer to as developmental synaptic degeneration [26–28] (see Results for details). Therefore, it is likely that these peripheral glial cells maintain developing newly-formed NMJs by providing signals to motor neurons and/or muscle cells. One attractive possibility is that Schwann cells secrete neurotrophic factors to preserve the nascent NMJ via promoting the survival of motor neurons (MNs) [29]. Alternatively or in addition, peripheral glial cells may stabilize nascent NMJs via other pathways, similar to central glia [30]. For example, perisynaptic Schwann cells modulate synaptic function at the postnatal NMJ [31,32], raising the possibility that embryonic Schwann cells maintain newly-formed NMJs by regulating activity-dependent signaling pathways in muscle and/or MNs. One family of activity-dependent molecules potentially regulated by Schwann cells at the NMJ are proteases. Administration of the broad-spectrum protease inhibitor leupeptin or the leech-derived antithrombin hirudin delayed synapse elimination at the postnatal NMJ [33,34]. More recent studies have shown a role for matrix metalloproteinases, neurotrypsin and calpains in regulating different aspects of neuromuscular synaptogenesis [35–37].

Here we provide genetic evidence that Schwann cells maintain nascent neuromuscular synapses by antagonizing the deleterious effects of peripheral neuromuscular activity. First, synaptic contact forms prematurely in *erbB3* mutant mice lacking Schwann cells, suggesting that alterations in signaling induced by synaptic activity may contribute to synaptic degeneration in these mutants. Consistent with this idea, developmental synaptic degeneration is completely blocked in *erbB3* mutants lacking ACh, muscle-derived ACh receptor, or evoked release of ACh, suggesting that MNs can survive in the absence of Schwann cell-derived neurotrophic factors. In order to identify molecular signals mediating this effect, we profiled gene expression in muscle with and without Schwann cells. We unexpectedly found that two serine protease inhibitors (serpins) classically viewed as anticoagulants, serpin C1 (i.e., antithrombin III) and serpin D1 (i.e., heparin cofactor II) are expressed in muscle-derived Schwann cells and down-regulated in muscle without Schwann cells. Because serpins C1 and D1 antagonize the activity of thrombin, a serine protease central to the hemostatic proteolytic cascade and with established cell signaling properties, we explored the impact of genetically imposed deficits of prothrombin on synaptogenesis in *erbB3* mutants. Remarkably, developmental synaptic degeneration was ameliorated when the *prothrombin* gene was deleted either in all cells or in a muscle-specific fashion or if the protease activated receptor-1 (PAR-1), a signaling receptor for thrombin, was inactivated. These results reveal that a protease traditionally associated with coagulation serves as a fundamental determinant of synaptogenesis and identify local thrombin signaling as a nexus of positive and negative modifiers derived from multiple NMJ components. To our knowledge, these studies provide the first genetic evidence that prothrombin derived from a local, non-hepatic cell is biologically meaningful. Together, these findings indicate a complex interaction between activity and glia that underlies the refinement and maintenance of developing neuromuscular synapses and have broader implications in understanding synaptic maintenance and treating neurodegenerative diseases [26–28].

## Results

### Genetic ablation of Schwann cells causes developmental synaptic degeneration

**Fig 1:**
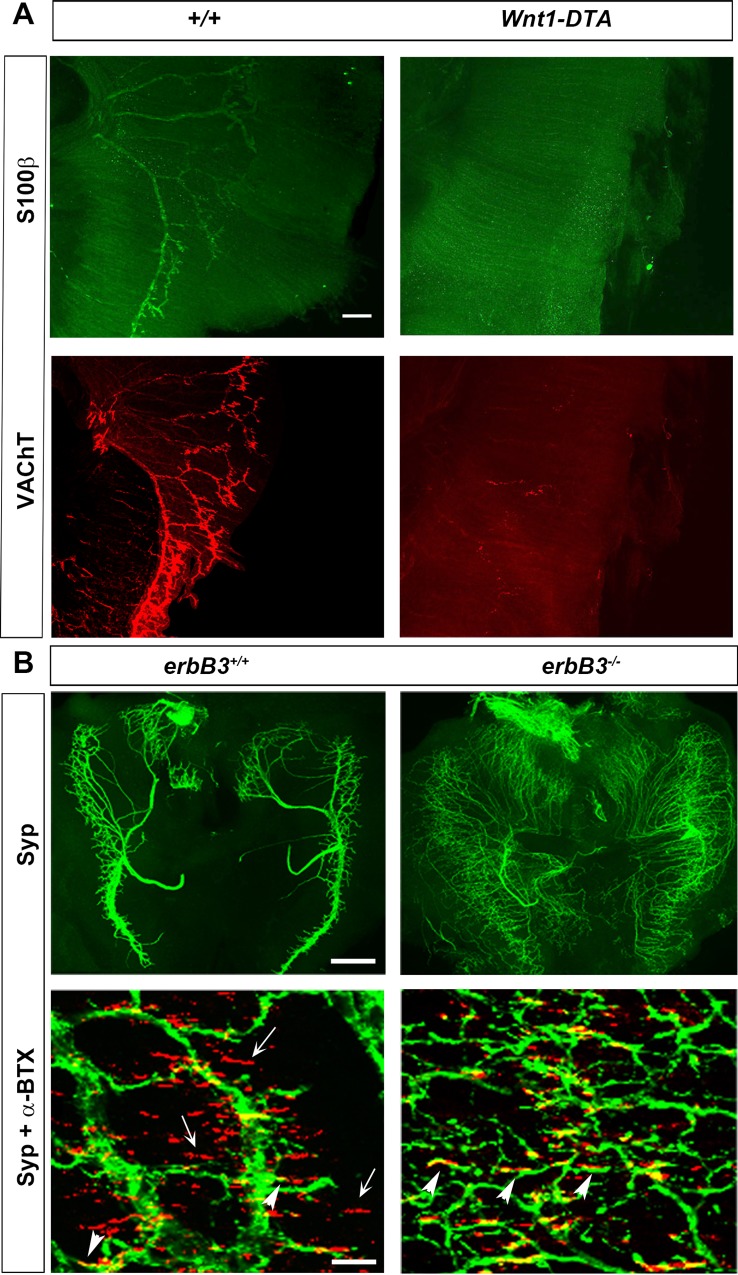
Developmental synaptic degeneration in the absence of Schwann cells. (A) Genetic ablation of Schwann cells, similar to *erbB* inactivation, leads to developmental synaptic degeneration of the neuromuscular junction (NMJ). Diaphragms were dissected at E15.5 from *Wnt1-Cre*:*Rosa26*^*LoxSTOPLox Diptheria Toxin A Chain*^ (*Wnt1-DTA*; right column) and wild-type mice (*+/+*; left columns), and stained with antibodies against S100 to label Schwann cells (green) and vesicular acetylcholine transporter (VAChT) to label presynaptic motor axon terminals (red). Scale bar = 70 μm. Representative example of *n* = 4. (B) Low- and high-power images (top and bottom panels, respectively) of diaphragm muscle at E14.25 show a strikingly higher percentage of α-bungarotoxin (α-BTX)-labeled postsynaptic nicotinic acetylcholine receptors (AChRs; red in bottom panels) receiving contact from synaptophysin-positive (Syp) motor axon terminals (green in top and bottom panels) in *erbB3* mutant (*erbB3*^*-/-*^; right panels) vs. wild-type (*erbB3*^*+/+*^; left panels) mice; arrows denote unapposed AChRs, arrowheads denote apposed or innervated AChRs. Scale bar in top panels = 250 μm; in lower panels = 50 μm.

Previous results showed that in the absence of NRG1 signaling, Schwann cells fail to migrate and proliferate along outgrowing peripheral nerves [20,25]. As a result, motor axons innervating the muscle target fail to travel in tightly associated bundles and instead appear defasciculated [21–25]. Nevertheless, nerve terminals transiently form synaptic contact in the appropriate endplate region of muscle fibers before swiftly undergoing degeneration between E14.5-E15.5 [22]. Because NRG1 and its erbB receptors are expressed in multiple cell types in the developing neuromuscular system, including MNs, Schwann and muscle cells, it is possible that the loss of NRG1 signaling in MNs and/or muscle, rather than the absence of Schwann cells, is responsible for developmental synaptic degeneration in *erbB2* or *erbB3* mutant mice. In order to address this possibility, we genetically ablated Schwann cells by crossing *Wnt1-Cre* mice to mice conditionally expressing the cytotoxic diphtheria toxin A-chain (*Wnt1-DTA*). Similar to *erbB2* and *erbB3* mutant mice, which display a complete absence of Schwann cells in the ventral roots as well as along the phrenic nerve and diaphragm motor endplates via histological, immunohistochemical and ultrastructural analysis [20,24]. *Wnt1-DTA* mice exhibit a near-complete loss of immunohistochemically detectable Schwann cells (3.8±3.8 S100-positive Schwann cells per hemi-diaphragm; *n* = 4). *Wnt1-DTA* mice also display a profound loss of motor axons innervating the diaphragm at E15.5, similar to *erbB* mutants, based on the absence of vesicular acetylcholine transporter (VAChT)-immunoreactive synaptic vesicles, which are transiently observed along developing motor axons [38] (7±3.2 VAChT-positive single motor axons per hemi-diaphragm; *Wnt1-DTA*; *n* = 4; **Fig 1A**). Thus, Schwann cell ablation caused by the absence of NRG1-mediated activation of erbB2/3 receptors on Schwann cells, rather than the loss of other NRG1 signaling pathways such as NRG1-mediated activation of erbB2/4 receptors on muscle cells [39], causes developmental synaptic degeneration at the NMJ.

### Aberrant synaptic contact in mutant mice lacking Schwann cells

In order to identify the molecular mechanisms underlying Schwann cell-mediated protection of NMJs, we examined the time course of developmental synaptic degeneration in the diaphragm muscle of *erbB3* mutant mice. At E14.25, NMJs were maximally innervated in *erbB3* mutant mice. At E14.75, nearly half of all NMJs exhibited neurofilament-labeled axon terminals with swellings or fractured, discontinuous immunolabeling (47±8%; 50 NMJs per diaphragm analyzed, *n* = 3). Between E15 to E15.5, nearly all synaptic boutons and phrenic motor axons had degenerated, with the few remaining axons exhibiting extensive fragmentation.

Interestingly, when *erbB3* mutant diaphragm was examined at E14.25, the last age at which the motor endplate retained complete innervation, we noticed a large increase in the proportion of α-bungarotoxin (α-BTX)-stained postsynaptic AChR clusters that were apposed to synaptophysin-immunoreactive presynaptic nerve terminals, when compared to wild-type (WT) diaphragm (**Fig 1B**). The percentage of synaptic contact observed in *erbB3* mutant mice at E14.25 was not detected in WT embryos until E16.0, nearly 2 days later (30.7 ± 11% vs. 89.3 ± 6.5% innervation, *P*< 0.005, E14.25 *erbB3* WT vs. mutant mice, 50 NMJs/ diaphragm; *n* = 3). It is possible that Schwann cells directly and tightly regulate synaptic contact. Alternatively, or in addition, the fasciculation of axons restricts their ability to navigate, and the loss of fasciculation caused by the absence of Schwann cells results in increased synaptic contact of axons or nerve terminals by chance. In either case, these results suggest that Schwann cells play a role in the initial timing of neuromuscular synaptic contact, in addition to their role in maintaining these synapses.

#### ACh elicits a muscle-derived retrograde signal to induce synaptic degeneration

**Fig 2:**
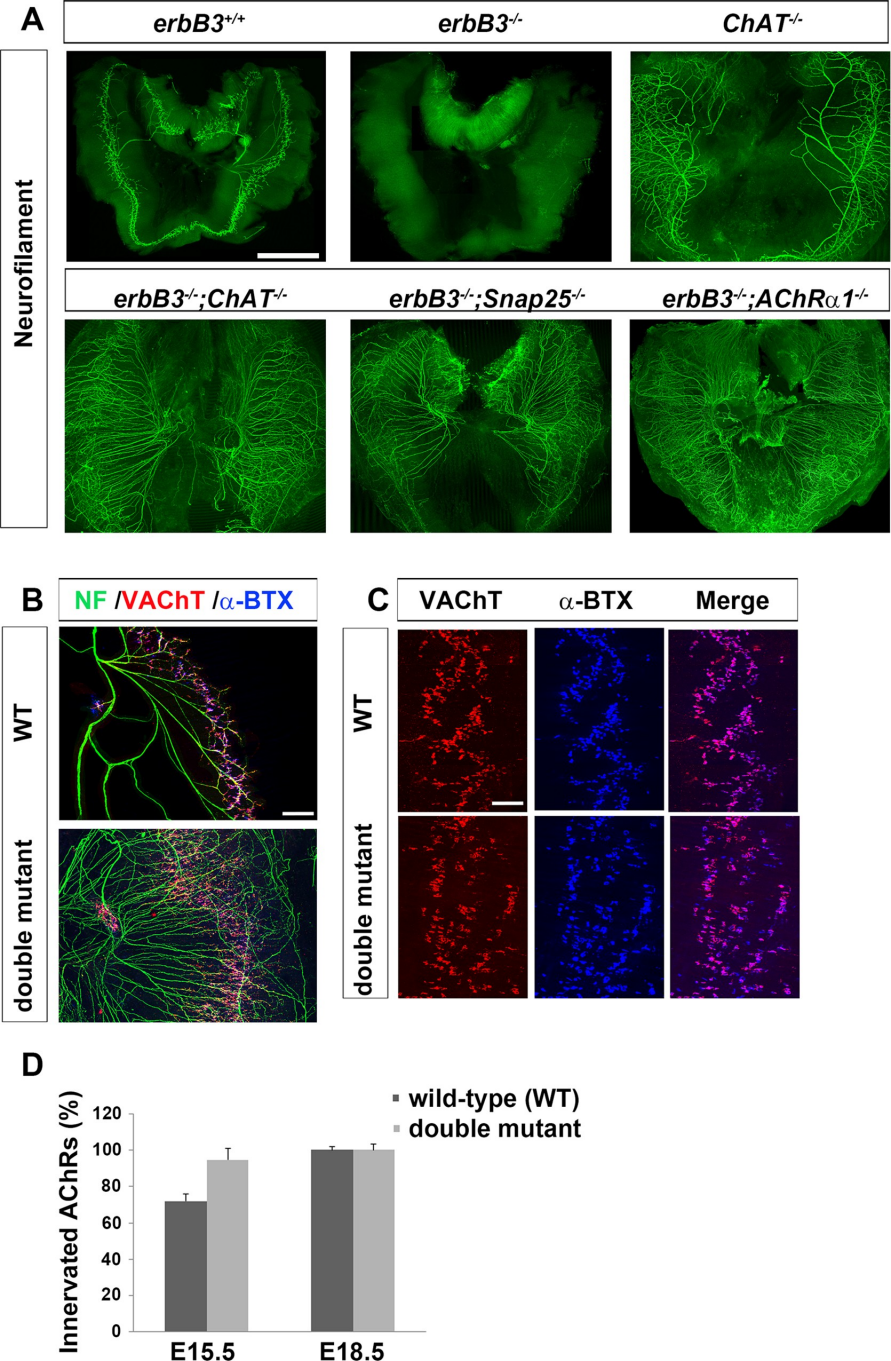
Evoked activity through muscle-derived AChRs is required for developmental synaptic degeneration induced by Schwann cell ablation. (A) E15.5 diaphragms from the indicated genotypes were dissected and immunostained with antibodies against neurofilament. In contrast to the absence of motor innervation observed in *erbB3* mutant (*erbB3*^*-/-*^) mice, *erbB3* mutant mice lacking ChAT, Snap25, or AChRα1 exhibit a complete lack developmental synaptic degeneration. The rescued axons are spread out as a consequence of the absence of Schwann cell-mediated fasciculation. Scale bar = 1000 μm. At least *n* = 3 for every genotype. (B) The motor endplate band is correctly positioned in the central region of *Snap25; erbB3* double mutant diaphragm. E15.5 diaphragms were labeled with antibodies against motor nerve (anti-neurofilament; NF; green), the presynaptic marker vesicular acetylcholine transporter (VAChT; red), and Cy5-α-BTX (blue). Scale bar = 150 μm. (C) Rescued synapses in E18.5 *Snap25; erbB3* double mutant diaphragm show apposition of VAChT-rich nerve terminals to postsynaptic, α-BTX-labeled AChRs. Scale bar = 100 μm, *n* = 3. (D) The relative number of VAChT-positive innervated AChRs is higher in E15.5 *erbB3; Snap25* double mutant vs. wild-type mice (similar to the increased innervation observed in single *erbB3* mutant vs. wild-type mice at E14.25), but is equal between these genotypes by E18.5 (*n* = 3 diaphragms for wild-type and double mutant mice, 50 NMJs counted per diaphragm).

It has been shown that repeated stimulation of muscle in chick increases the degeneration of MNs [40]. Because neuromuscular synaptic contacts formed precociously in *erbB3* mutant mice, we hypothesized that synaptic activity may contribute to developmental synaptic degeneration in Schwann cell-deficient mice. In order to test this idea, we examined *erbB3* mutant mice lacking choline acetyltransferase (ChAT), the biosynthetic enzyme for ACh. *ChAT* mutants exhibit no spontaneous or evoked ACh release[3]. In striking contrast to *erbB3* mutant diaphragm, which is largely devoid of motor axons at E15.5 (5.4±1.1 vs. 0.6±0.55 neurofilament-positive secondary phrenic nerve branches, *erbB3* WT vs. *erbB3* mutant mice; *P*<0.0005; *n* = 5), *erbB3*;*ChAT* double mutant diaphragm contains more axons (5.4±1.1 vs. 98±22.3 neurofilament-positive secondary phrenic nerve branches, *erbB3* WT vs. *erbB3;ChAT* double mutant; *n* = 3; **Fig 2A**). The increase of secondary branches in the diaphragm of double mutants likely reflects a combination of the increase of motor axons caused by the blockade of cell death observed in the *ChAT* single mutants [41,42] (**Fig 2A**) together with the defasciculation of motor axons observed in the *erbB3* single mutants. Similar results were observed in *erbB2*;*ChAT* double mutant diaphragm (**S1 Fig**). These results show that developmental synaptic degeneration induced by Schwann cell deletion is prevented by the absence of neural activity.

Next, we sought to determine whether nerve-evoked or spontaneous release of ACh is required for developmental synaptic degeneration by eliminating *Snap25* in *erbB3* mutants, which resulted in a loss of evoked but not spontaneous release of ACh [43]. Similar to *ChAT* mutants, mice lacking both *erbB3* and *Snap25* exhibit a complete rescue of motor axons and innervated NMJs (5.4±1.1 vs. 102.3±25 neurofilament-positive secondary phrenic nerve branches, *erbB3* WT vs. *erbB3;Snap25* double mutant; *n* = 3; **Fig 2A**), suggesting that evoked activity is required for triggering this degenerative process. The presynaptic terminals of rescued motor axons in *Snap25*;*erbB3* double mutant diaphragm terminated onto postsynaptic α-BTX-labeled AChRs within a centrally located endplate band, similar to WT NMJs, at E15.5 (**Fig 2B**), as well as at E18.5 (97.7±2.5% vs. 97.7±3.2% VAChT-positive, α-BTX-labeled AChR clusters, *erbB3* WT vs. *erbB3;Snap25* double mutant; *n* = 3; **Fig 2C and 2D**). Finally, in order to examine whether the evoked release of ACh mediates its effect on muscle-derived AChRs, rather than motor axon-derived AChRs, we took advantage of *AChRα1* mutant mice. The α1 subunit is restricted to muscle, mediates the regressive effects of ACh on muscle, and is required for the assembly of the AChR complex in muscle [44]. Similar to the effects of the removal of Snap25 and ChAT, genetic inactivation of AChRα1 in *erbB3* mutant mice prevented developmental synaptic degeneration (5.4±1.1 vs. 91±19.5 neurofilament-positive secondary phrenic nerve branches, *erbB3* WT vs. *erbB3; AChRα1* double mutant; *n* = 3; **Fig 2A**), suggesting that evoked neurotransmission through muscle AChRs is required for the deleterious effects of activity on the maintenance of NMJs in Schwann cell-deficient muscle. Taken together, these results suggest that the evoked release of ACh, acting through muscle-derived AChR, induces a negative retrograde signaling pathway that triggers developmental synaptic degeneration in the absence of Schwann cells.

### Endogenous thrombin inhibitors are expressed by Schwann cells

In order to identify muscle-derived, activity-regulated factors that might induce this negative signaling pathway, we performed transcriptome analysis on diaphragm samples isolated from *erbB3* WT and mutant mice at E14.75, when maximal denervation was observed. We performed experiments on two different diaphragm samples and found that 240 and 152 genes were upregulated and 242 and 240 genes were downregulated in each *erbB3* WT vs. mutant muscle sample (**S2A and S3A Figs).** Expression of Schwann cell-specific markers such as *Sox10* and myelin protein zero (*MPZ*) were reduced in *erbB3* mutant relative to WT muscle (0.5% and 2.4% by RPKM; Reads Per Kilobase Million), further corroborating the absence of Schwann cells and their associated RNAs in *erbB3* mutant muscle (**S2B Fig**).

**Fig 3:**
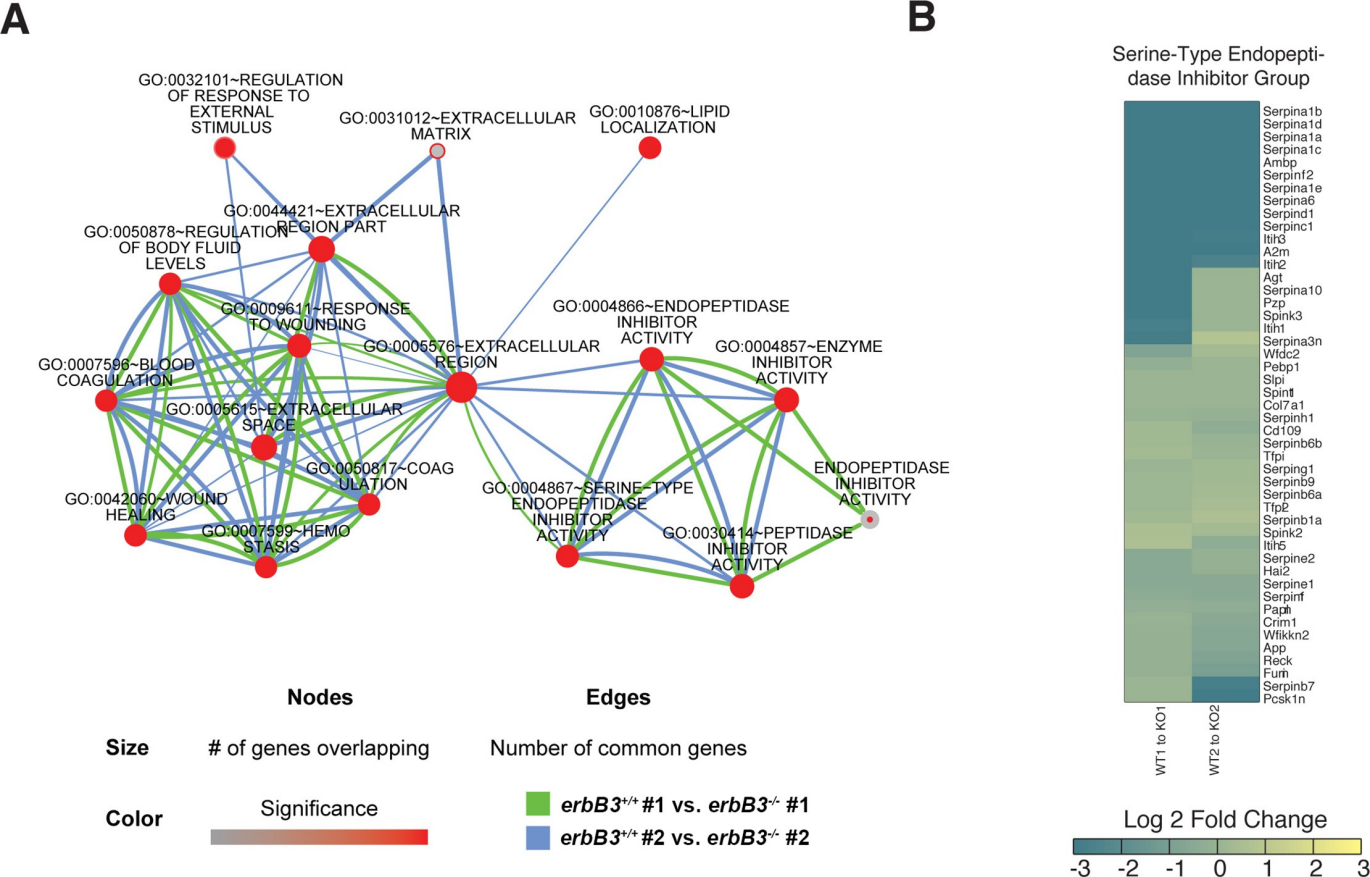
Functional genomic analysis of genes differentially regulated in diaphragm muscle containing (wild-type; *erbB3*^*+/+*^) or lacking (mutant; *erbB3*^*-/-*^) peripheral Schwann cells. (A) Gene Ontology (GO) term networks from the results of GO analysis of the set of genes significantly upregulated in *erbB3* wild-type vs. mutant muscle were overlapped in Cytoscape. Two individual comparisons (each one between *erbB3* wild-type- and mutant-derived muscle samples) were performed and are represented by the blue and green lines. The number of gene members of each term, and degree of overlap, or genes common, between multiple terms are represented by node and edge attributes. Highly interconnected nodes with overlap from both comparisons are particularly noteworthy. (B) Heatmap showing fold-changes in gene expression of some individual members of the GO terms differentially regulated in *erbB3* wild-type and mutant muscle. Expression values were determined by the number of mapped reads normalized to gene length and depth of sequencing.

We used DAVID (Database for Annotation, Visualization and Integrated Discovery) to perform gene ontology (GO) analysis for biological process (BP), cellular component (CC) and molecular function (MF) on differentially expressed genes in each of the two comparisons between *erbB3* WT and mutant muscle (**Table 1** and **S3B and S3C Fig**). Significantly different terms of BP, CC or MF were then compared using Cytoscape in order to visualize the consistency between each of the two comparisons as well as the overlap between the terms themselves. We found that gene products differentially expressed in muscle of *erbB3* WT vs. mutant mice were often associated with wound healing, coagulation and serine protease inhibition (**Fig 3A**). Several transcripts were downregulated in *erbB3* mutant muscle and, interestingly, two of them encode classic inhibitors of the procoagulant serine protease thrombin, namely antithrombin III (serpin C1) and heparin cofactor II (serpin D1) (**Fig 3B**). The specificity of these serpin expression changes was illustrated by the fact that a third serpin with antithrombin potential, protease nexin I (PN-1; serpin E*2*), was not differentially expressed (**Fig 3B**). Previous *in vitro* studies showed that thrombin is expressed by muscle and causes motor neuron death [45,46]. Therefore, we hypothesized that the loss of thrombin inhibition, caused by the reduction of antithrombin expression in *erbB3* mutant muscle, drives developmental synaptic degeneration in these mice.

**Table 1:** Functional categories of genes upregulated in muscle in the presence of Schwann cells.

	***erbB3***^***+/+***^ **#1** **vs.** ***erbB3***^***-/-***^ **#1** ***(*****240)**			***erbB3***^***+/+***^ **#2** **vs.** ***erbB3***^***-/-***^ **#2** **(152)**		
**GO Categories**	**Category #s**	**Term**	**Corrected *P* value**	**Category #s**	**Term**	**Corrected *P* value**
Molecular Function	GO:0030414	Peptidase inhibitor activity	4.48E-27	Endopeptidase inhibitor activity	GO:0004866	1.25E-08
	GO:0004866	Endopeptidase inhibitor activity	3.25E-26	Serine-type endopeptidase Inhibitor activity	GO:0004867	2.63E-08
Cellular Component	GO:0005576	Extracellular region	1.24E-24	Extracellular region part	GO:0044421	4.76E-17
	GO:0005615	Extracellular space	4.62E-19	Extracellular region	GO:0005576	5.42E-17
Biological Process	GO:0009611	Response to wounding		Response to wounding	GO:0009611	0.009146505
	GO:0050817	Coagulation				

Examples of the most significantly enriched Gene Ontology (GO) terms in the list of upregulated genes in WT vs. erbB3 mutant muscle, as annotated. The *P* value indicated was corrected for multiple testing using the Benjamini-Hochberg me

We first verified expression differences of serpins C1 and D1 in *erbB3* WT vs. mutant muscle by qRT-PCR (**S2D Fig**). To obtain direct evidence of Schwann cell-specific serpin C1 and D1 expression, we employed a cell-specific profiling technique to probe the muscle-derived Schwann cell transcriptome. *Ribotag* mice expressing a Cre-dependent, hemagglutinin (HA) epitope-tagged Rpl122 ribosomal protein [47] were crossed to *Wnt1-Cre* mice. We confirmed via immunohistochemistry that the HA epitope was robustly expressed by phrenic nerve-associated Schwann cells in the diaphragm at E14.75 (**S4A Fig**). Next, we isolated ribosome-associated mRNAs from diaphragm muscle of these mice and performed RNA-Seq (*n* = 2; see methods for details). Raw sequencing reads clearly showed an abundance of Sox10 in muscle samples derived from *Wnt1-Ribotag* and *erbB3* WT but not *erbB3* mutant mice (**S4B Fig**). Serpins C1 and D1 were also observed in muscle samples derived from *Wnt1-Ribotag* mice (**S4C Fig**). Together, these studies show that Schwann cells in the diaphragm express serpins C1 and D1 at an age at which MNs require Schwann cells for synaptic maintenance.

**Fig 4:**
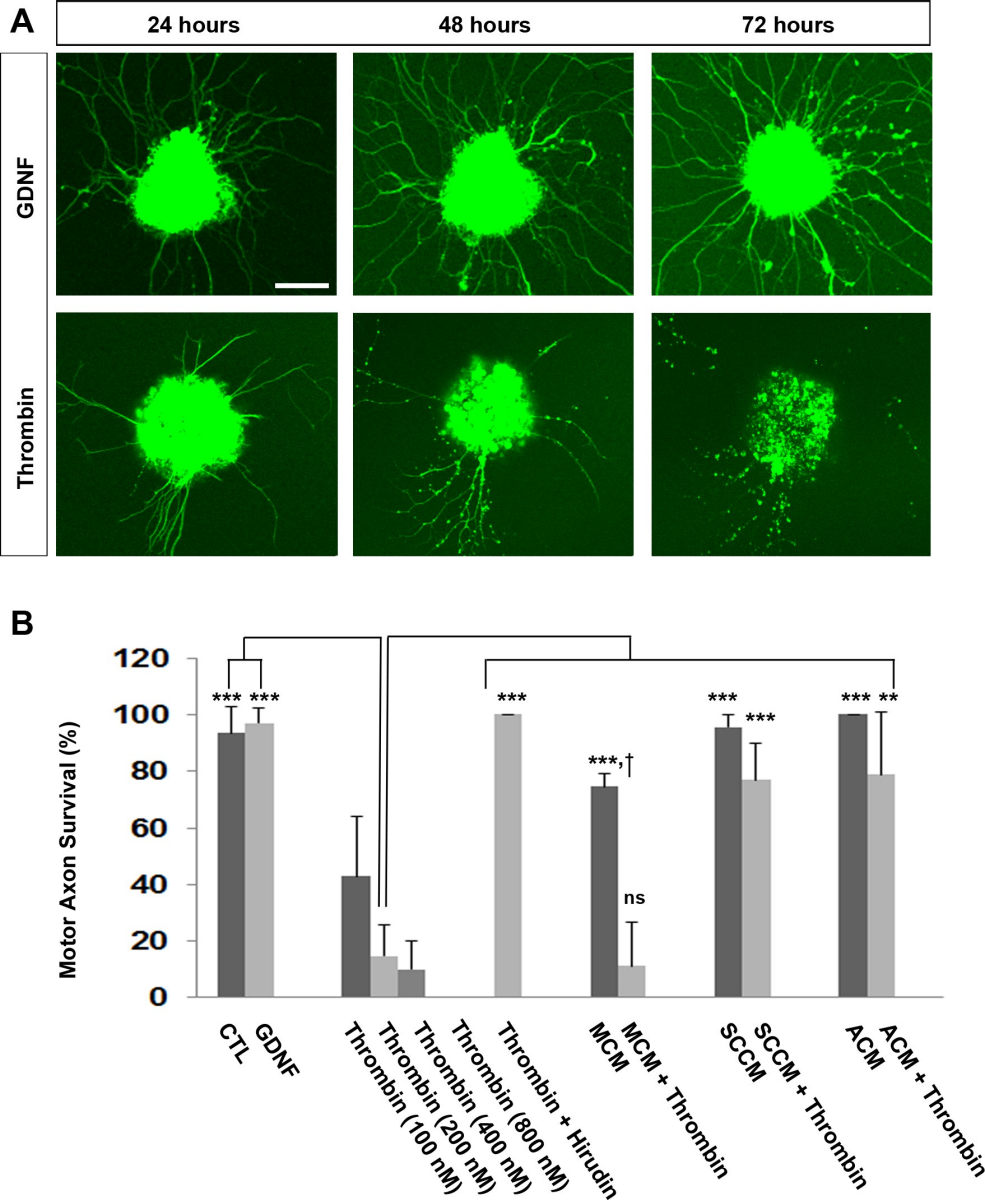
Thrombin causes motor axon degeneration *in vitro* and is blocked by pre-incubation with glia- but not muscle-conditioned medium. E12.5 cervical spinal explants from *HB9*:*GFP* mice were grown on laminin, treated at plating with 5 nM GDNF in B27-containing neurobasal (B27-NB) medium, and re-imaged 24 hours after specific treatments, and the number of GFP-positive motor axons with pathological swelling or other signs of degeneration were quantified at pre- and post-treatment intervals. (A) Representative images of explants each treated with GDNF at plating and then treated with GDNF (top panels) or 200 nM recombinant thrombin (lower panels) one day after plating. Images were captured one day after plating (left column), and one and two days after treatment (middle and right panels, respectively). Scale bar = 200 μm (B) Quantification of axon degeneration, represented by the percentage of degenerating motor axons observed one day after vs. before treatment. Thrombin exerted a dose-dependent increase in the number of degenerating motor axons, which was significantly different from that in control, GDNF-treated, muscle-conditioned medium (MCM)-, Schwann cell-conditioned medium (SCCM)-, and astrocyte-conditioned medium (ACM)-treated explants (***, *P*<0.005, *n* = 3). Pre-incubation for 15 minutes of 200 nM thrombin with hirudin (at 500 μg/mL), SCCM, or ACM, but not MCM (not significant; ns), blocked the degenerative effects of thrombin on motor axons (***, *P*<0.005, **, *P*<0.001). Treatment with MCM alone produced a significantly higher number of degenerating axons vs. CTL or GDNF treatment (cross, *P*<0.005).

In order to determine whether Schwann cell-derived endogenous antithrombins were capable of blocking the degenerative effects of thrombin *in vitro*, we modified a MN explant outgrowth assay [48]. We cultured cervical spinal cord explants prepared from E12.5 *HB9*:*GFP* mice (expressing GFP in motor axons) with the growth factor GDNF for one day, imaged the explant, and then further cultured these explants in either control, GDNF- or thrombin-containing media for an additional 24 hours prior to re-imaging. Thrombin exerted a dose-dependent response, inducing the degeneration of nearly all motor axons at 200 nM (**Fig 4A**). Pre-incubation of thrombin for 15 minutes with control media or serum-free media that was conditioned by differentiated C2C12 muscle cells for 24 hours failed to block the degenerative effect of thrombin on motor axons *in vitro* (**Fig 4B**). In contrast, when thrombin was pre-incubated with primary astrocyte- or primary Schwann cell-conditioned media, the negative effects of thrombin were potently inhibited. A similar protective effect from thrombin-induced degeneration was found by pretreatment with the thrombin-specific inhibitor from leech, hirudin (**Fig 4C**). Based on the specificity and known mechanism of action of hirudin, these *in vitro* studies demonstrate that thrombin proteolytic activity (and not merely prothrombin) is a determinant of developmental synaptic degeneration.

### Muscle-derived prothrombin induces developmental synaptic degeneration through PAR-1

**Fig 5:**
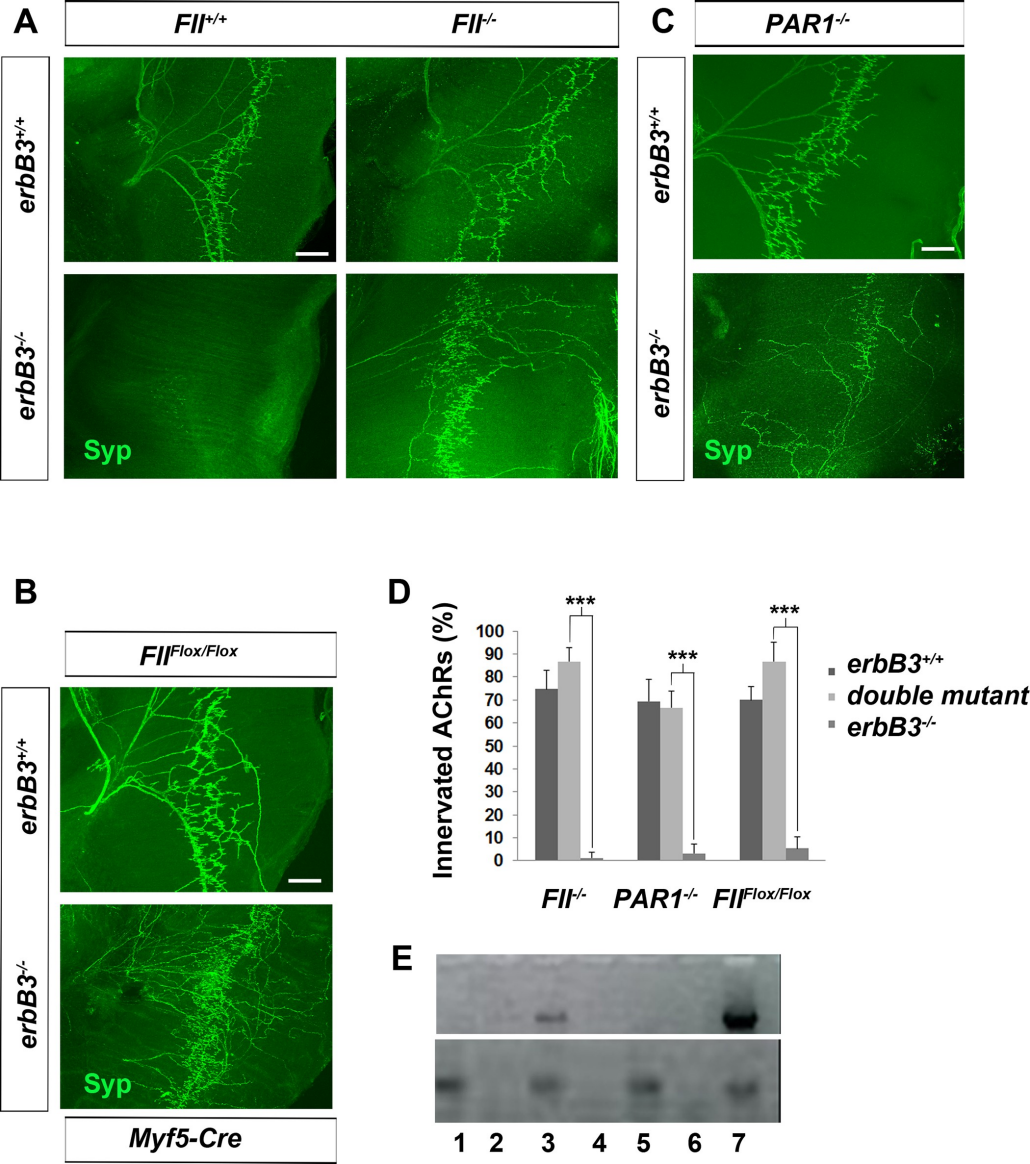
PAR-1 mediates thrombin-induced developmental synaptic degeneration caused by Schwann cell ablation. (A) E15.5 diaphragms from the indicated genotypes were dissected and immunostained with antibodies against synaptophysin (green). Note the retention of motor innervation of NMJs in *prothrombin* (*FII*), *erbB3*;*FII* double mutant (*erbB3*^*-/-*^*; FII*^*-/-*^; bottom right panel) vs. *erbB3* single mutant diaphragm (*erbB3*^*-/-*^; bottom left panel). In contrast, there is no difference in the motor innervation between prothrombin wild-type (*FII*^*+/+*^) and mutant (*FII*^*-/-*^) diaphragm (top panels). (B) Muscle-specific elimination of prothrombin in *erbB3* mutants (*erbB3*^*-/-*^; *FII*^*Flox/Flox*^; *Myf5-Cre*; bottom panel) results in the rescue of presynaptically innervated NMJs. Scale bar in A-C = 100 μm. (C) *PAR1*; *erbB3* double mutant diaphragm (*erbB3*^*-/-*^*; PAR1*^*-/-*^; bottom panel) also exhibits a rescue of motor innervation, whereas *PAR1* single mutant diaphragm is similar to that of *PAR1* wild-type (top panel). (D) Quantification of NMJs. The percentage of α-BTX-labeled AChRs apposed to synaptophysin-immunoreactive presynaptic terminals in diaphragm muscle is significantly higher in *erbB3* mutants lacking *FII*, *PAR1* or muscle-derived *FII* when compared to single *erbB3* mutants alone (*erbB3*^*-/-*^; *FII*^*Flox/Flox*^; *Myf5-Cre* vs. *erbB3*^*-/-*^; ***P<0.0005, *n* = 3 diaphragms for each genotype). (E) Lack of muscle-derived prothrombin/FII expression in *FII*^*Flox/Flox*^; *Myf5-Cre* mice, 1,2 = muscle from *FII*^*Flox/Flox*^; *Myf5-Cre* mice (+,- reverse transcriptase; RT); Lanes 3,4 = muscle from *Myf5-Cre* and *FII*^*Flox/+*^; *Myf5-Cre* mice (+,- RT); Lanes 5,6 = muscle from *FII*^*-/-*^ mice (+,- RT); Lane 7 = liver from wild-type mice (+RT). β-actin expression from same samples is shown below.

In order to determine if the loss of Schwann cell-associated antithrombins, including serpins C1 and D1, is responsible for developmental synaptic degeneration in *erbB3* mutant muscle, we examined the effect of genetic deletion of the *prothrombin* gene on synaptic degeneration in *erbB3* mutants. Constitutive prothrombin deficiency results in a loss of vascular integrity and an embryonic lethal phenotype in most, albeit not all embryos, by E10.5 [49,50]. This partial embryonic lethal phenotype provided the opportunity to investigate the role of thrombin at the NMJ in embryos that lacked Schwann cells. We found a striking preservation of motor innervation in prothrombin-deficient *erbB3* mutant diaphragm, compared to *erbB3* mutant littermates with one or two copies of the *prothrombin* gene (5.2±1.3 vs. 34.7±9.3 synaptophysin-positive secondary phrenic nerve branches, *erbB3* WT vs. *erbB3;prothrombin* double mutant; *n* = 3; 72.3±10.3% vs. 82.7±5% synaptophysin-positive clusters, *erbB3* WT vs. *erbB3;prothrombin* double mutant, at least 50 NMJs counted per diaphragm, *n* = 3; **Fig 5A**). Deletion of prothrombin alone had no effect on motor innervation of the diaphragm, suggesting that thrombin does not prune axon branches or NMJs in the presence of Schwann cells, but triggers the degeneration of these same synapses in the absence of Schwann cells. Intriguingly, the rescue of motor innervation in *erbB3* mutant mice lacking prothombin is not as complete as in those lacking evoked activity, suggesting that, in addition to prothrombin, other factors may contribute to developmental synaptic degeneration. Along these lines, we examined the role of pro-brain-derived neurotrophic factor, which is stimulated by activity and induces refinement of Xenopus neuromuscular synapses through activation of the neurotrophin receptor p75 [51], but failed to detect the rescue of developmental synaptic degeneration in the *p75*; *erbB3* double mutant mice (**S5A Fig**).

The predominant sources of prothrombin during embryonic development are the yolk sac and liver [52]. Because glia are known to regulate permeability of the blood-brain barrier in the CNS and blood-nerve barrier in the periphery [53], one possible way by which thrombin may trigger the loss of motor innervation is through an increase of vascular permeability in peripheral Schwann cell-deficient muscle, which would allow egress into the nerve of systemic molecules such as thrombin. However, when we examined vessel structure of the diaphragm by PECAM1 immunostaining or vessel integrity by FITC dextran staining after transcardial injection, we were unable to observe any overt differences between *erbB3* WT and mutant diaphragm at E14.25, before the onset of denervation (**S5B Fig**). On the other hand, previous findings suggest that skeletal muscle cells express and secrete active thrombin [34,45,54]. In order to genetically test this idea, we took advantage of conditional *prothrombin* mutants [55]. Similar to the effects of constitutive deletion of prothrombin, conditional deletion in muscle of prothrombin in an *erbB3* WT background failed to affect the pattern or number of innervated NMJs at E15.5. However, the NMJs of *erbB3* mutant mice lacking prothrombin in muscle exhibited strikingly preserved motor innervation compared to *erbB3* mutant littermates expressing one or two copies of the *prothrombin* gene in muscle (5.2±1.3 vs. 44.7±10.5 synaptophysin-positive secondary phrenic nerve branches, *erbB3* WT vs. *erbB3;*conditional *prothrombin* double mutant; *n* = 3; **Fig 5B and 5D**).

Prothrombin is unlikely to mediate the regressive effects of activity in the presence of Schwann cells, because the number of axon branches and synapses is similar between WT and constitutive or conditional *prothrombin* mutants (**Fig 5A**), whereas it is greater in *ChAT* mutants [41,42]. One potential explanation underlying this observation is that in the presence of Schwann cells, prothrombin is not expressed, whereas in the absence of Schwann cells, prothrombin is induced. However, prothrombin is expressed at similar levels in *erbB3* WT and mutant diaphragm (**S6A Fig**). Alternatively, the activation of prothrombin to thrombin by enzymes such as coagulation factor 10a (factor Xa) or fibrinogen-like protein 2 (fgl2) may be induced by the absence of Schwann cells. Although the expression of the gene encoding factor Xa, factor X, was not reliably detected by RNA-Seq at E14.75, fgl2 was not differentially expressed in these samples (0.5 ± 0.2 vs. 0.6 ± 0.064 RPKM, *erbB3* WT vs. mutant, *P* = 0.51, *n* = 2). However, when we examined factor X and fgl2 expression by qPCR at E14.25, we observed enhanced levels of factor X in *erbB3* mutant diaphragm lacking Schwann cells (15.1±2.1 vs. 19.7±1.6 fold-change relative to WT adult, E14.25 *erbB3* WT vs. mutant, *P* = 0.036, *n* = 3), but no change in fgl2 or prothrombin levels. These data suggest that Schwann cells may normally prevent the activation of prothrombin to thrombin by regulating the expression of factor X. In order to determine if factor X is developmentally regulated, similar to prothrombin [34], we evaluated its expression in the diaphragm endplate region of WT mice at E14.25, P15, and adult. Similar to prothrombin, we found that factor X expression is developmentally regulated, with higher expression occurring at E14.25 and P15, relative to adult (**S6B Fig**). In contrast, the expression of serpins C1 and D1 in the endplate region of the diaphragm were not significantly different at each of these timepoints (serpin D1: 1.24 ± 0.15 vs. 1.16 ± 0.07 vs. 1.04 ± 0.08 fold-change relative to WT adult, E14.25 vs. P15 vs. adult; serpin C1: 1.19 ± 0.12 vs. 1.27 ± 0.06 vs. 1.1 ± 0.1 fold-change relative to WT adult, E14.25 vs. P15 vs. adult).

We next explored how neuromuscular activity regulates the response of Schwann cell-deficient NMJs to muscle-derived prothrombin. While the expression levels of serpins C1 and D1 were indistinguishable in *erbB3* mutant muscle with or without activity, prothrombin levels were markedly reduced in *erbB3* mutant muscle lacking activity (**S6A Fig**). In order to determine if inactivity exerts the same effects on prothrombin protein expression as on gene expression in muscle, we examined C2C12 myotubes stimulated with the ACh agonist carbachol. Using an antibody specific to mouse prothrombin and thrombin [50], we observed a marked induction of both prothrombin and thrombin in the conditioned medium of carbachol-stimulated C2C12 cells, whereas treatment with the voltage-gated sodium channel blocker tetrodotoxin reduced the expression of both inactive and active thrombin (**S6C Fig**). Together, these studies show that the blockade of neural transmission at the NMJ results in a potent downregulation of prothrombin expression, which accounts for at least a portion of the protective effects exerted by inactivity in Schwann cell-deficient muscle.

**Fig 6:**
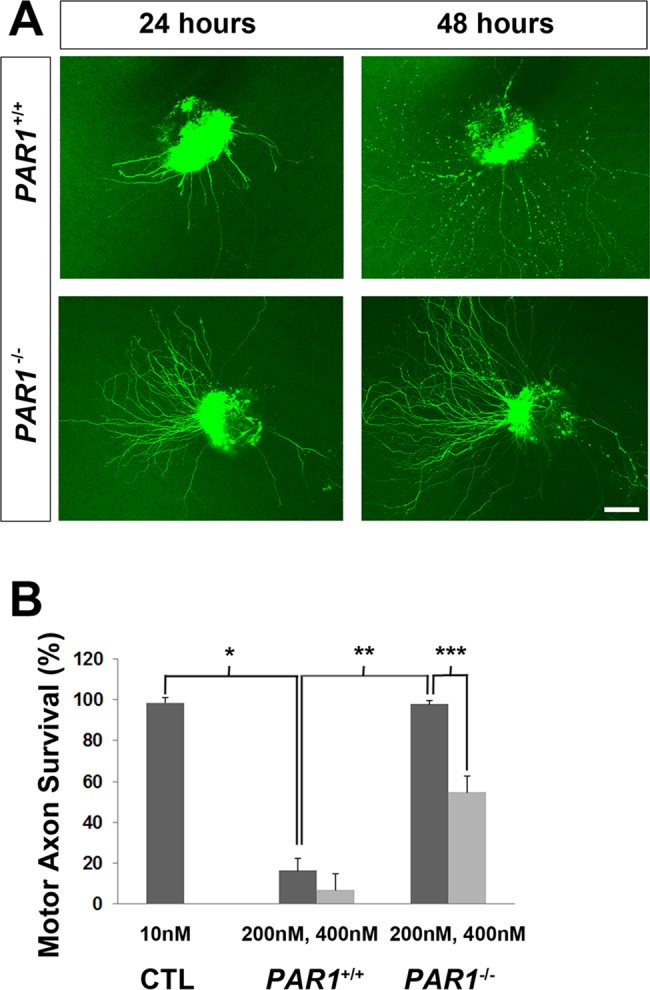
Thrombin fails to cause motor axon degeneration in spinal explants derived from *PAR1* mutant mice. (A) Explants obtained from *PAR1* wild-type; *HB9*:*GFP* (*PAR1*^*+/+*^, top panels) and *PAR1* mutant; *HB9*:*GFP* mice (*PAR1*^*-/-*^; bottom panels) were plated with 10 nM GDNF and imaged the next day (left panels). Explants were then treated with 200 nM thrombin and imaged a second time the following day (right panels). In contrast to explants of *PAR1* wild-type mice, which exhibit axonal degeneration in response to 200 nM thrombin, those of *PAR1* mutant mice are largely unaffected. Scale bar = 100 μm. (B) The protective effect of PAR-1 deletion on thrombin-mediated axonal degeneration is dose-dependent, because higher concentrations of thrombin (400 nM) elicit motor axon pathology. CTL refers to GDNF treatment at plating and again at 1 day after plating. Each value reflects the percentage of healthy motor axons at 2 vs. 1 day after plating, and represents the mean of 3 samples. *P<0.0005, thrombin-treated vs. control; **P<0.0005, thrombin-treated *PAR1*^*-/-*^ vs. thrombin-treated *PAR1*^*+/+*^*explants;* ***P< 0.0005, 200 nM vs. 400 nM thrombin-treated *PAR1*^*-/-*^ explants. Student’s *t* with Bonferroni correction.

Thrombin exerts its biological function in part via the cleavage of the N-terminal region of the protease-activated receptor-1 (PAR-1), a G-protein coupled receptor, to generate a tethered auto-ligand which in turn activates PAR-1 downstream pathways [56]. PAR-1 is a member of the PAR family that also includes PAR-2-4. We added activating peptides of PAR-1 and PAR-4 to HB9:GFP explants, since PAR-2 is not activated by thrombin and PAR-3 merely supports PAR-1 signaling [56,57]. We found that PAR-1 but not PAR-4 activating peptides could mimic thrombin-mediated degeneration of motor axons *in vitro* (**S7 Fig**). In order to examine whether MNs themselves express PAR-1, we compared the effects of thrombin on HB9-GFP motor explants prepared from *PAR1* WT and mutant mice. In contrast to those from *PAR1* WT mice, motor explants derived from *PAR1* mutant mice [58] exhibited resistance to thrombin-induced degeneration (**Fig 6**), suggesting that MN-derived PAR-1 expression is required. Although detection of PAR-1 protein with antibodies is complicated by the absence of specific antibodies, the *PAR1* mutants also express the *lacZ* gene, thus allowing for the determination of cellular expression. When we examined the muscle of these mice, AChR-innervating motor axons were robustly labeled with antibodies against the *lacZ* gene product β-galactosidase (**S8 Fig**), further supporting the idea that muscle-derived thrombin acts directly on MN-derived PAR-1. Finally, we crossed *PAR1* mutants to *erbB3* mutants and observed a preservation of motor innervation similar to that of *erbB3* mutants lacking constitutive or muscle-derived prothrombin (5.2±1.3 vs. 29.7±3.1 synaptophysin-positive secondary phrenic nerve branches, *erbB3* WT vs. *erbB3;PAR1* double mutant; *n* = 3; **Fig 5C and 5D**). These findings demonstrate that MN-derived PAR-1 mediates muscle-derived, thrombin-induced developmental synaptic degeneration in Schwann cell-deficient muscle.

## Discussion

**Fig 7:**
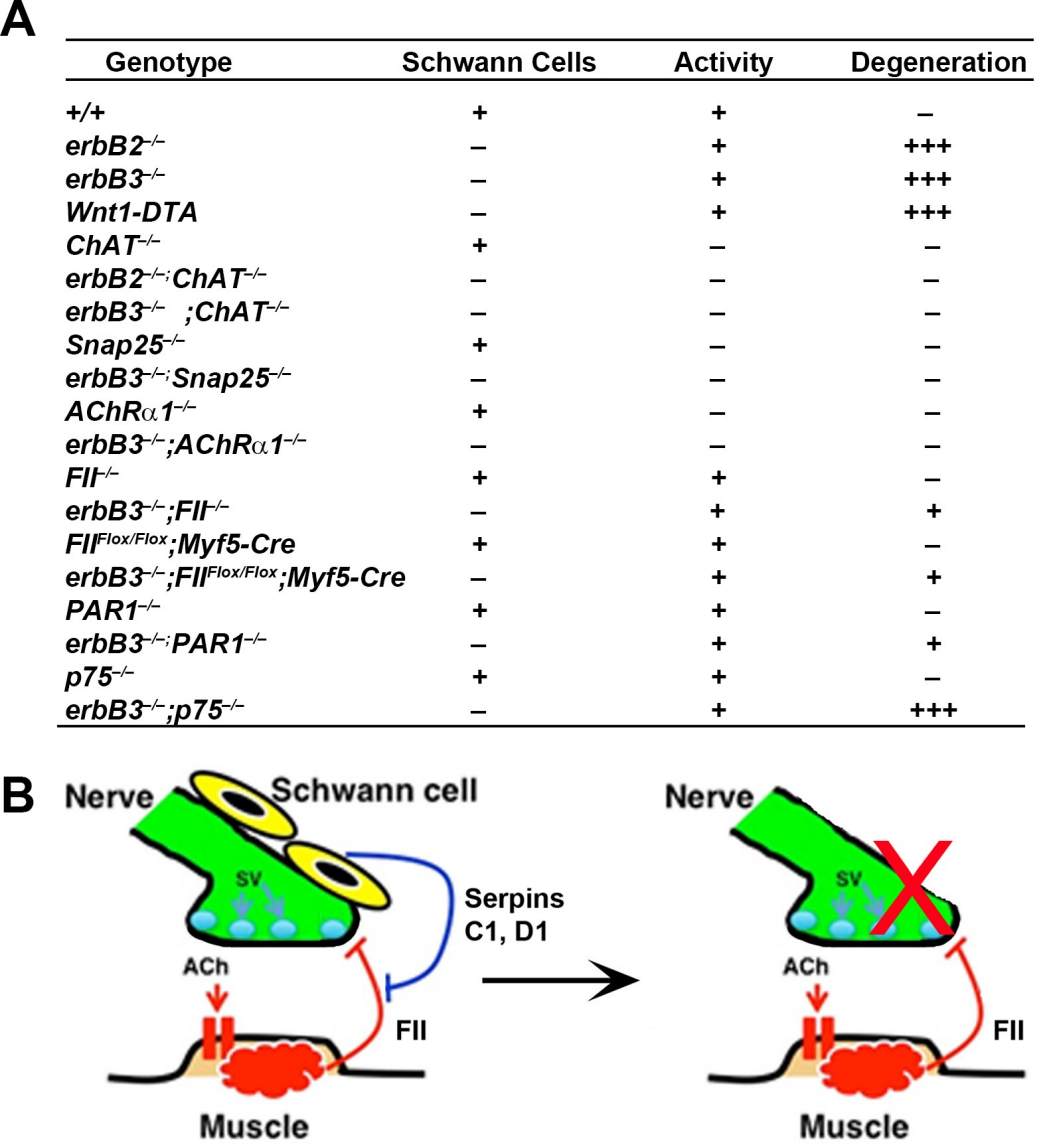
Model for the interplay of neural activity and Schwann cells in maintaining the motor innervation of the NMJ. (A) Summary of the presence or absence of Schwann cells, neuromuscular activity (+,–), or developmental synaptic degeneration in 19 mouse lines. *erbB2*^*-/-*^ mice refer to *erbB2* null mutants crossed to transgenic mice overexpressing *erbB2* in the heart, which survive until birth[20]. (B) (Left panel) Presynaptic nerve terminals (green) release ACh onto muscle-derived AChRs, resulting in the release of muscle-derived thrombin (factor II; FII), whose activity is normally opposed by serpins C1 and D1 released from Schwann cells. (Right panel) In mice lacking Schwann cells, thrombin causes developmental synaptic degeneration (red X) because of the absence of these antithrombins.

Based on these (**Fig 7A**) and previous findings [34,46,59,60], we propose the following model for the role of Schwann cells in the formation and maintenance of the motor innervation of developing neuromuscular synapses (**Fig 7B**). First, Schwann cells regulate the timing of initial synaptic contact between muscle and nerve. Next, nerve-derived ACh induces the expression in muscle of prothrombin, which is released and activated before acting in a retrograde fashion to trigger the degeneration of presynaptic motor axon terminals. Finally, Schwann cells prevent the activation of prothrombin to thrombin by downregulating the expression of factor X and antagonize muscle-derived thrombin by expressing serpins C1 and D1. Thus, Schwann cells antagonize the effects of neural activity indirectly by inhibiting the degenerative effects of muscle-derived negative signals. This model provides a framework for considering several aspects on the mechanisms underlying the interplay of neural activity and glial cells in regulating synaptic maintenance in development and in disease.

First, these results identify new regressive signaling events induced by neural activity at the embryonic NMJ. For example, neural activity destabilizes postsynaptic AChR clusters. These anterograde effects of ACh are inhibited by nerve-derived agrin [9], similar to the inhibition of the retrograde effects of ACh by Schwann cell-derived antithrombin described in this study. However, in the absence of agrin, ACh causes destabilization of postsynaptic AChRs but not degeneration of presynaptic boutons and axons, whereas in the absence of Schwann cells, ACh causes presynaptic degeneration but not postsynaptic destabilization. Thus, ACh elicits distinct negative signaling pathways in muscle to coordinate different aspects of synaptogenesis. While these results suggest that neuromuscular activity eliminates the motor innervation of NMJs lacking Schwann cells in part through a retrograde thrombin pathway, additional pathways downstream of activity are likely, since elimination of thrombin is not as effective or persistent as elimination of activity itself. Some of these pathways may be dependent on the normal function of muscle, which undergoes significant atrophy, protein catabolism and impaired growth in response to disuse or other forms of reduced activity [61].

Second, these results highlight the role played by glia in regulating the relationship between neural activity and synaptic maintenance. For example, in mouse models of motor neuron disease, neurotransmitter release and transmitter-mediated activation of terminal/perisynaptic SCs are increased presymptomatically in a mouse model of amyotrophic lateral sclerosis (ALS) [62]. These cells also exhibit structural alteration in mouse models of spinal muscular atrophy [63], suggesting that the early dysregulation of synaptic function in these diseases may lead to the loss of synaptic maintenance through alterations in Schwann cell signaling.

These results also suggest that local sources of thrombin may trigger synaptic degeneration in various pathological contexts involving damage to glia. In such a scenario, the ability of glial cells to antagonize thrombin activity would be diminished. For example, Schwann cells lacking antithrombin PN-1 (serpin E2) exhibit a delay in functional recovery after nerve injury [64]. Similarly, consistent with expression of thrombin inhibitors in central glia [65,66], thrombin was identified as a pathological component of plaques in multiple sclerosis (MS), a disease of central axon-associated glia [67], and may serve as a potential early biomarker for MS [68]. Additionally, thrombin accumulation has been observed in the brains of patients with Alzheimer’s disease [69], suggesting that glia-derived antithrombin signaling may be compromised, either directly by disease-causing proteins or indirectly by changes in neural activity.

Thrombin may also play a role in the reduction of synapses that is observed in schizophrenia, as dysregulated expression of complement proteins, whose levels are regulated by thrombin signaling [70,71], is associated with an enhanced susceptibility to this disease [72]. These findings may also provide insight into the mechanisms underlying the finding that chronic warfarin therapy unexpectedly causes remission of psychotic symptoms in schizophrenia [73]. Together with recent evidence showing that the terminal complement pathway (i.e., starting with the generation of C5a from C5 by C5 convertase or thrombin [70]) is upregulated and promotes the degeneration of NMJs in ALS mouse models [74], these data also suggest that chronic warfarin therapy may be neuroprotective in the context of ALS. Together, these studies point to glial cells as an important integrator of synaptic function and maintenance and suggest that therapies aimed at restoring glial function may help prevent synaptic degeneration and thereby maintain synapses in disease.

## Methods

### Mice

*erbB3* mutant mice were kindly provided by Genentech [24]. *erbB2*, *ChAT* and *AChRα1* mutant mice were described previously [20,41,44]. Constitutive and conditional prothrombin mutants were described previously [49,55]. *HB9*:*GFP* mice were kindly provided by Sam Pfaff (The Salk Institute). *PAR1* mutant mice were purchased from mutant mouse regional resource center (MMRRC; Davis, CA [55]). *Snap25* mutant, *Ribotag*, *Myf5-Cre*, *Wnt1-Cre*, *Rosa26-LSL-Tomato* and *Rosa26-LSL-DTA* mice were all purchased from the Jackson Lab (Bar Harbor, ME). The use of animals is in compliance with the guidelines of the Animal Care and Use Committee of the Salk Institute.

### RNA isolation, RNA-Seq, and real-time quantitative PCR

For whole muscle or muscle endplate samples, total RNA was isolated from embryonic or postnatal diaphragm muscle in Trizol reagent (Invitrogen). Briefly, diaphragms, or the endplate region surrounding the phrenic nerves, were minced into small pieces and then passed through a 20-guage needle five times in 1 mL of Trizol, allowed to sit on ice for 5 minutes, then passed similarly through 22-, 23- and 25-guage needles, before extraction. For Ribotag samples [47], an entire litter of diaphragms produced from a cross between a homozygous Ribotag mouse and a transgenic *Wnt1-Cre* mouse was dissected and quickly homogenized in polysome buffer by needle as above. Genotyping subsequently revealed half of these embryos to have been Cre-positive and hemizygous for the Ribotag allele. We analyzed two of these pooled samples (i.e., each sample represents 4 pooled *Wnt1-Cre*, *Ribotag* diaphragms taken from a litter). After immunoprecipitation and RNA elution using Promega RNA MicroEasy buffers, RNA was evaluated with a PicoQuant chip on a BioAnalyzer. The RNA Integrity number was above 8 and the concentration was ~150–200 pg/ml. For RNA-seq, 500 ng (whole muscle) or 2 ng (Riobtag sample) of RNA was incubated with oligodT beads (TruSeq, Illumina) and incubated at 65°C for 5 minutes to enhance mRNA binding to beads. Beads were rinsed and eluted for two minutes at 80°C, and eluants allowed to re-bind beads. Purified mRNA was then fragmented (300–500 bp) and primed with random hexamers at 95°C for 8 minutes. mRNA was then reverse transcribed into the first strand of cDNA and then fragmented using a Bioruptor sonicator. After second strand cDNA synthesis, double stranded cDNA libraries were end-repaired, adenylated, and ligated with indexed adapter primers to facilitate library amplification and sequencing. Libraries were amplified 11 cycles based on analysis with SYBR Gold staining, and PicoGreen quantitation (Molecular Probes/Invitrogen) was used to determine final library concentrations. For real-time qPCR, samples were treated with DNase (Invitrogen) before reverse transcription with Superscript III (Invitrogen) and Oligo(dt) primers. cDNAs from samples were amplified and detected using SYBR Green I reagent (Roche) and a LightCycler 480 Instrument (Roche), or on a BioRAD CFX Connect. Quantification of mRNA levels was performed using the LightCycler 480 Software (Roche), which calculates the expression ratio using an efficiency-calibrated method [75]. Target mRNA levels were normalized to expression of β-actin. Primer sequences are listed in **S10 Fig**. For timecourse studies, the gene expression fold-changes were normalized to adult samples. Unpaired Student’s *t* tests were used in statistical analysis.

### Explants and cell culture schwann cells

E12.5 *HB9*:*GFP* embryos were collected and placed into 1X Hank’s balanced salt solution (HBBS) on ice [76]. The roof plate of the spinal cord was opened before removal from the vertebral column, and following removal the meninges were pulled briskly off of the spinal cord. This open-book preparation of the cord was then pinned onto Sylgard-coated 35 mm petri dishes filled with B27-containing, L-glutamine supplemented Neurobasal (B27-NB) medium, and the region of cord lateral and medial to the motor columns were excised by micro-dissecting knife. Motor columns from the cervical region were then cut transversely using micro-iridectomy scissors into explants and were transferred into poly-d-lysine pre-coated (PDL; 1 μg/ml in borate solution, overnight), laminin-coated (5 μg/ml if glass, 1 μg/ml if plastic) coverslips placed into 4-well trays or into 48-well tissue culture trays. Explants were plated in B27-NB supplemented with 5 ng/ml glial-derived neurotrophic factor (GDNF; R&D). Images of GFP-positive motor axons arising from these explants were taken at approximately the same time the following day on an Olympus FluoView 1000 confocal microscope at 10X or 20X magnification, gently rinsed, changed to pre-incubated NB-B27 plus experimental treatment, and then imaged the following day. The percentage of healthy axons devoid of pathological swellings were measured and presented as percent survival at 24 hours and 48 hours after plating (i.e., 24 hours after treatment). Unpaired Student’s t-tests with the Bonferonni correction were used for statistical analysis.

C2C12 myoblasts were cultured on 2% gelatin-coated 6-well tissue culture trays in DMEM with 20% fetal bovine serum (FBS). When confluent, the culture medium was changed to differentiation medium, which was DMEM with 2% normal horse serum (NHS). For experiments analyzing prothrombin/thrombin levels by immunoblotting, 5-day differentiated cultures were rinsed and replaced with 1.5mL serum-free DMEM. One day later, cultures were treated with saline, the acetylcholine agonist carbachol (CCh; 100 μm; Sigma) or the sodium channel blocker tetrodotoxin (TTX; 10 μM; Enzo Life Sciences). 18 hours following treatment, conditioned medium was collected, passed through a 0.2-μm filter, and spun through a 3kD cutoff column, before being eluted and mixed with equal volume 2X Laemmli buffer (62.5 mM Tris, 20% glycerol, 2% SDS, and 5% 2-mercaptoethanol). Lysates were rinsed in cold phosphate-buffered saline (PBS, pH 7.3) and then lysed in RIPA buffer with sodium fluoride, sodium orthovanadate, and protease inhibitors, sonicated, centrifuged, and supernatants mixed with Laemmli buffer before boiling. For experiments analyzing the effects of conditioned medium on spinal explants, 5-day differentiated cultures were rinsed and replaced with serum-free NB-B27, and 1 day later, the medium was removed, passed through a 0.2-μm filter, and administered without concentration to explants with or without co-treatments (e.g., thrombin).

Astrocyte cultures were prepared from E17.5 mouse cortex. After dissection, removal of meninges, and chopping into small blocks, tissue from one brain was treated with 10 ml of 0.25% trypsin (Worthington) in HBSS for 5 minutes, passed through a 5 ml syringe, and incubated in trypsin 2–3 more minutes. Trypsin was replaced with 1 ml of 20% FBS/DMEM DMEM, and tissue was gently triturated 3 times with a 5-ml syringe, allowed to float to bottom, and supernatant was transferred to a new tube. One ml of fresh 20% FBS/DMEM was added and the process was repeated 2 more times. Similar trituration with fire-polished glass pipets was performed 3 more times, producing roughly 6 ml of cell suspension, which was subsequently passed through a 70-μm filter and spun at 1000 rpm for 2 minutes. The medium was removed and the pellet was re-suspended with 10% FBS/DMEM supplemented with penicillin/streptomycin and added to a PDL-pre-coated 100-mm petri dish. The following day, cells were rinsed 3X in warmed DMEM and replaced with fresh growth medium (10% FBS/DMEM). 6–8 days after plating or when cells approached confluence, the tray was tapped extensively to liberate microglia, rinsed, trypsinized in 0.25% trypsin-EDTA for five minutes and re-plated in growth medium onto PDL-coated 6-well dishes. Two days after re-plating, medium was rinsed and replaced with serum-free NB-B27 and 1 day later, the medium was removed, passed through a 0.2-μm filter, and administered without concentration to explants with or without co-treatments (e.g., thrombin).

Schwann cell cultures were prepared from P1-P2 neonatal sciatic nerves using a procedure based on Wei et al. [77] with slight modification. Both nerves from one animal were dissected from the ventral and dorsal cord (with care to remove spinal ganglia) to the knee. The nerves were incubated in 0.3% collagenase type II for 30 minutes at 37°C, switched into 0.25% trypsin-EDTA for 5 minutes at 37°C, and resuspended into 10% FBS/DMEM growth medium supplemented with penicillin and streptomycin. After centrifugation at 100 rpm for 2 minutes, resuspended cells (from 2 nerves of 1 animal) were plated into a single PDL-coated 60-mm petri dish. The following day, the medium was replaced with 2% FBS/DMEM supplemented with 10 ng/mL NRG1 (R&D) to facilitate the growth of Schwann cells but not fibroblasts. Two days later, cultures were rinsed and replaced with 2% FBS/DMEM supplemented with 10 μM cytosine arabinoside to kill fibroblasts. Two days later, cells were treated with 0.05% trypsin for 1 minute to differentially remove Schwann cells but not fibroblasts from the plate, and these cells were spun down and re-plated onto PDL-coated 6-well dishes, treated with NRG1 for 2–4 more days, replaced with serum-free NB-B27 and 1 day later, the medium was removed, passed through a 0.2-μm filter, and administered without concentration to explants with or without co-treatments (e.g., thrombin).

### Immunohistochemistry

Diaphragm muscles were fixed in 2% paraformaldehyde (PFA) in 0.1 M phosphate buffer (pH 7.3) overnight at 4°C, rinsed briefly with PBS, incubated in 0.1 M glycine in PBS for 1 h, rinsed briefly with PBS and then washed with 0.5% Triton X-100 in PBS. The muscles were blocked with 10% FBS and then incubated with primary rabbit antibodies against neurofilament-150 (1:1000, Millipore), synaptophysin (1:1000; Santa Cruz), S100 (1:1000; DAKO), β-gal (1:1000, MP Biomedicals) or MuSK (1:1000, gift of L. Mei, Case Western Reserve University, OH), guinea pig antibodies against VAChT (Millipore), mouse anti-hemagglutinin (HA) antibodies (Covance), or incubated with Alexa-Fluor-488-conjugated fasciculin to label AChE (2 μg/ml, kind gift of R. Rotundo, University of Miami, FL) in blocking buffer overnight at 4°C. After being washed three times for 1 h each in 0.5% Triton X-100 in PBS, the muscles were incubated with fluorescent secondary antibodies and/or Cy2- or Cy3- or Cy5-conjugated-α-BTX (1:1500, Molecular Probes) overnight at 4°C.

### Western blotting

Rabbit anti-prothrombin antiserum was kindly provided by Evan Sadler (Washington University, MO). Extracts of mouse hindlimb, diaphragm or liver, or lysates or conditioned media concentrates of differentiated C2C12 muscle cells, were prepared in RIPA buffer, sonicated, diluted in 2X Laemmli buffer and boiled 5 minutes at 100°C.

## Supporting information

S1 Fig Developmental synaptic degeneration is rescued in *erbB2* mutant mice lacking activity. *erbB2* mutant mice were crossed to *ChAT* mutant mice (right panels). Diaphragms were dissected at E17.5 and stained with neurofilament antibody (green). Scale bar = 1000 μm.
(TIF)

S2 Fig Differentially regulated genes identified by RNA-seq analysis and confirmed by qPCR. (A) Scatter-plots reveal genes (circles) that are significantly upregulated (red) or downregulated (green) in *erbB3* wild-type (*+/+*) vs. mutant (*-/-*) muscle. Each plot represents a separate biological sample. (B) Analysis of RNA sequencing tracks confirmed that Schwann cell-specific genes such as Sox10 and Myelin Protein Zero levels were reduced in *erbB3* mutant muscle (*-/-* #1,*-/-* #2) to 0.5% and 2.4%, respectively, of values derived from wild-type muscle (*+/+* #1, *+/+* #2). (C) Functional genomic analysis of genes differentially regulated in diaphragm muscle containing (*erbB3* wild-type; +/+) or lacking (*erbB3* mutant; *-/-*) peripheral Schwann cells. (C) Gene Ontology (GO) term networks of the set of genes significantly upregulated in *erbB3* mutant vs. WT muscle, overlapped in Cytoscape. The pathways most highly upregulated in *erbB3* mutant muscle were related to muscle contractility. In contrast to the upregulation of serpins in muscle from WT mice, there was an increase of serine protease expression in *erbB3* mutant muscle.
(TIF)

S3 Fig Transcriptomic sequencing analyses. (A) Number of raw and mapped reads in each of two diaphragm samples from *erbB3* wild-type (*+/+* #1, *+/+* #2) vs. mutant (*-/-* #1, *-/-* #2) mice at E14.75, as well as comparison of the number of upregulated and downregulated genes between each pair of samples derived from *erbB3* wild-type and mutant mice. (B) Gene ontology categories most highly upregulated in *erbB3* wild-type sample 1 vs. mutant sample 1 and (C) *erbB3* wild-type sample 2 vs. mutant sample 2 show that serine protease inhibitors are highly expressed in wild-type muscle containing Schwann cells vs. *erbB3* mutant muscle lacking Schwann cells. (D) qPCR analysis shows that expression of the serpins D1 and C1 are 10-fold and 6-fold higher, respectively, in diaphragm muscle derived from *erbB3* wild-type vs. mutant mice at E14.75, whereas expression of serpin E2 is unchanged. Fold-changes are relative to changes in β-actin expression. Dotted line indicates normalized expression of genes in *erbB3* mutant muscle. Each value represents (*n* = 3), samples run in duplicate.
(TIF)

S4 Fig Schwann cell transcriptome screen of diaphragm muscle at E14.75 exhibits expression of serpins C1 and D1. (A) Staining of diaphragm muscle derived from *Wnt1-Ribotag* (*Wnt1-Cre*; *Rpl22*^*LoxSTOPLox Ribotag*^) mice at E14.75 with a monoclonal antibody against hemagglutinin (HA) shows robust expression of epitope-tagged ribosomes in Schwann cells along the phrenic nerve. Scale bar = 10 μm. (B) Raw sequencing tracks of *Sox10* in diaphragm samples at E14.75 derived from *erbB3* mutant mice (Rows 1–2), from WT mice (Rows 3–4), and from *Wnt1-Cre*, *Ribotag* mice (Rows 5–6). (C) Reads per kilobase per million mapped read (RPKM) values from muscle-derived samples of the indicated genotypes for the Schwann cell markers *Sox10* and myelin protein zero (*MPZ*) as well as for the anti-thrombins *serpinC1* and *serpinD1*. The enrichment of *Sox10* and *MPZ* in Schwann cells, as determined by *Wnt1-Ribotag* RPKMs, is higher than for *serpinC1* and *serpinD1*, which may indicate that these proteins are expressed by both muscle and Schwann cells.
(TIF)

S5 Fig Inactivation of the proBDNF receptor p75 fails to inhibit developmental synaptic degeneration in *erbB3* mutant diaphragm. (A) *erbB3* mutant (*-/-*) mice were crossed to *p75* mutant (*-/-*) mice, embryos were sacrificed at E15.5, and diaphragm muscles were stained with antibodies against synaptophysin. Scale bar = 100 μm. *n* = 3 for *erbB3; p75* double mutants. (B) Diaphragm muscles from E14.25 *erbB3* wild-type (+/+) and mutant (*-/-*) mice were imaged after immunostaining with PECAM1 antibodies (red, top panels) or after transcardial injection of FITC dextran (70-kDa, green, bottom panels). Scale bar = 50 μm.
(TIF)

S6 Fig Thrombin but not antithrombin is regulated by activity in muscle. (A) qPCR analysis shows that while expression of the *serpins D1* and *C1* is significantly higher in wild-type vs. *erbB3* mutant (*-/-*) muscle at E14.75, expression of prothrombin (Factor II or FII) is unchanged. In contrast, whereas *serpinD1* and *serpinC1* expression levels in muscle are unchanged by inactivity (i.e., equal expression in *erbB3*^*-/-*^ vs. *erbB3*^-/-;^*ChAT*^-/-^ mice), *prothrombin* expression is significantly reduced by inactivity. *P<0.005, *serpinC1* and *serpinD1*, *erbB3* wild-type vs. mutant mice. **P<0.001, *prothrombin*, *erbB3*^*-/-*^ vs. *erbB3*^-/-;^*ChAT*^-/-^ mice. Fold-changes are relative to changes *in β-actin* expression. Dotted line indicates normalized expression of genes in *erbB3* mutant muscle. Each value represents (*n* = 3), samples run in duplicate. (B) Developmental timecourse of *prothrombin* and *factor X* gene expression by qPCR in the endplate region of the diaphragm. Fold-changes are relative to changes *in β-actin* expression and normalized to the level of *prothrombin* and *factor X* expression in adult samples. Each value represents (*n* = 3), samples run in duplicate. (C) Western analysis shows that cholinergic stimulation of muscle cells leads to an increase of prothrombin and active thrombin protein in the conditioned medium. Top and bottom panels reflect the same gel cut in half and show prothrombin and active, cleaved thrombin immunoreactivity, respectively. Whereas thrombin immunoreactivity is observed at approximately 25 kD based on loading of recombinant thrombin (bottom panel, lane 1), prothrombin immunoreactivity is detected near 75 kD (arrow), based on loading of muscle extracts from prothrombin wild-type and mutant mice at E14.75 (*FII*^*+/+*^ and *FII*^*-/-*^; top panel, lanes 2 and 3). Note the absence of a band at this molecular weight in prothrombin mutant muscle. Treatment of differentiated, C2C12 muscle cells with the ACh agonist carbachol (CCh) increased the amount of prothrombin (top panel, lane 6) and active thrombin (bottom panel, lane 6) found in the medium, compared to activity-blocked cultures (both panels, lanes 4 and 5).
(TIF)

S7 Fig PAR-1-activating peptide (PAR1-AP) mimics the effects of thrombin on motor axon degeneration *in vitro*. (A) qPCR analysis shows that expression of PAR-1, PAR-3 and PAR-4 is unchanged in the ventral spinal cord at E14.75 of *erbB3* wild-type vs. mutant mice. Fold-changes are relative to changes in β-actin expression. Dotted line indicates normalized expression of genes in *erbB3* mutant muscle. Each value represents (*n* = 3), samples run in duplicate. (B) PAR1-AP, at a concentration of 100 μM, but not PAR4-AP, causes significant degeneration of *HB9*:*GFP*-positive motor axons when administered 1 day after plating with 5 nM GDNF. CTL refers to 5 nm or 10 nM GDNF treatment at plating and again 1 day after plating. Each value reflects the percentage of healthy motor axons at 2 vs. 1 day after plating, and represents the mean of 3 samples. Dark grey bars = lower dose and light grey bars higher dose of agent. *P<0.01, Student’s *t* with Bonferroni correction. Scale bar = 200 μm.
(TIF)

S8 Fig PAR-1 expression is detected in motor neurons. Hindlimbs from *PAR1* mutant mice expressing LacZ (*PAR1*^*LacZ/LacZ*^) at E14.75 were sectioned and stained with antibodies against β-galactosidase (β-gal; red) and fluorescent α-BTX (green). Note the staining of motor axons innervating α-BTX-labeled AChRs. Scale bar = 20 μm.
(TIF)

S9 Fig Normal positioning of endplate band in *erbB3* mutants lacking thrombin / PAR1. Diaphragm muscles from samples in Fig 5 stained both with synaptophysin as well as with α-bungarotoxin (α-BTX) show the normal central positioning and size of the endplate band of nicotinic AChR clusters. Scalebar = 100 μm.
(TIF)

S10 Fig qPCR primer sequences. Sequences of primers used to detect expression of beta-actin, prothrombin, factor X, fgl2, serpin C1 and serpin D1 via qPCR, and PCR product lengths.
(TIF)

S1 Data Raw data for results presented only in the text (row 1–12) or presented in figures (rows 17–28 and 31–41). For each set of results, the age, genotype and dependent variable are given, as well as averages, standard deviations and statistical tests, are provided.
(XLSX)
