# Supplementary figures and images for: Glial cells maintain synapses by inhibiting an activity-dependent retrograde protease signal
Source: PLoS Genet. 2019 Mar 14;15(3):e1007948. doi: 10.1371/journal.pgen.1007948 (PMC6417855; doi:10.1371/journal.pgen.1007948)

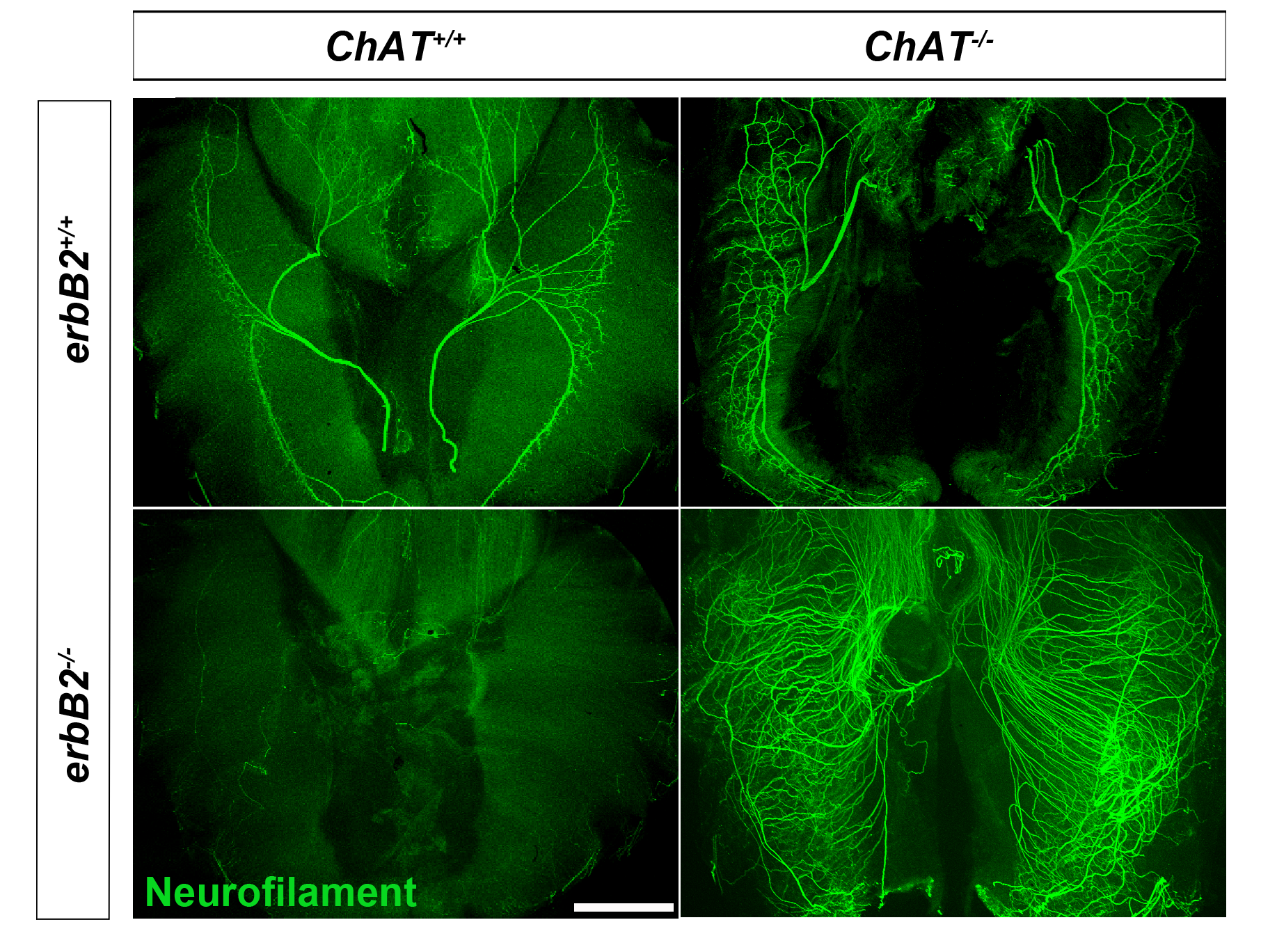

Supplement: S1 Fig — erbB2 mutant mice were crossed to ChAT mutant mice (right panels). Diaphragms were dissected at E17.5 and stained with neurofilament antibody (green). Scale bar = 1000 μm. (TIF) [file pgen.1007948.s001.tif]

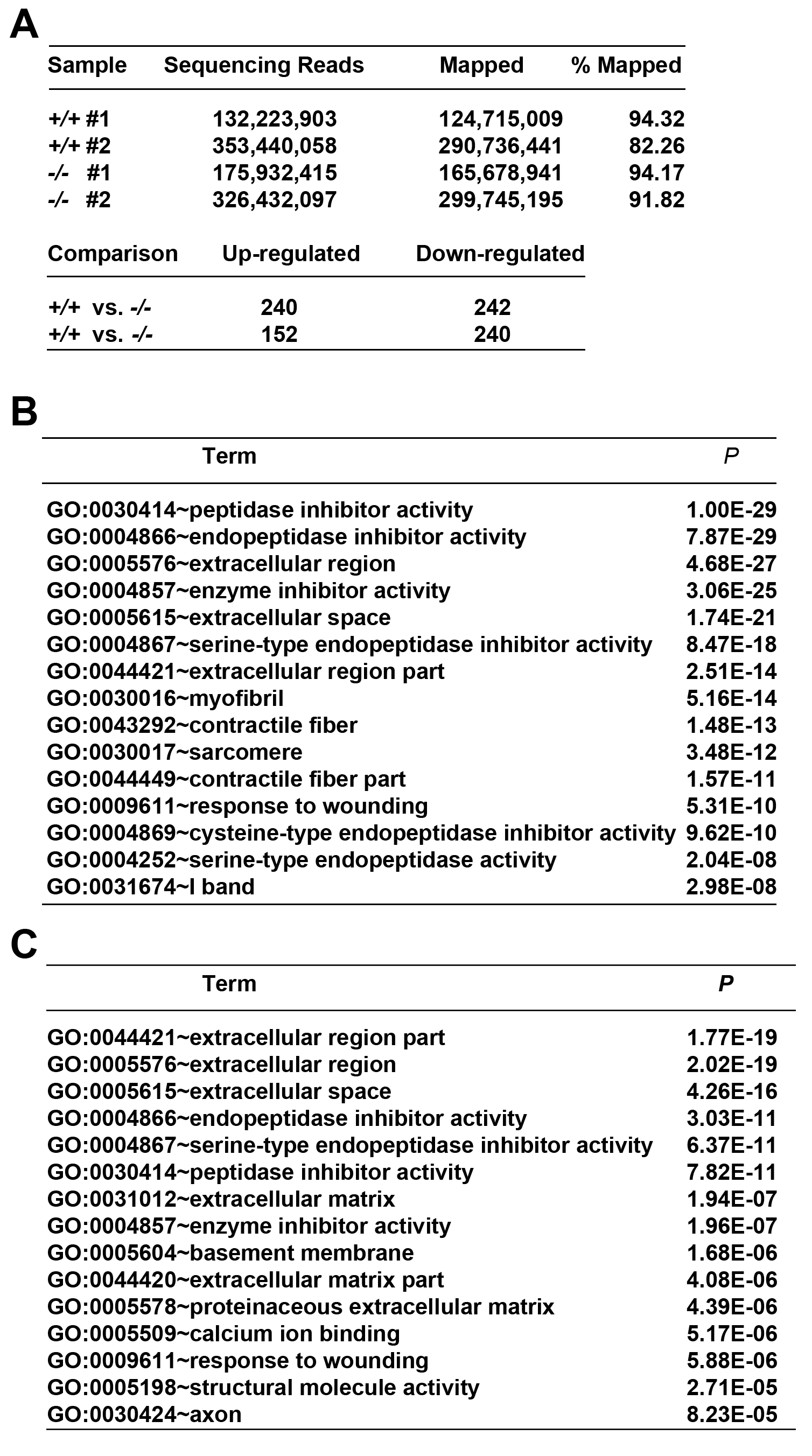

Supplement: S2 Fig — (A) Scatter-plots reveal genes (circles) that are significantly upregulated (red) or downregulated (green) in erbB3 wild-type (+/+) vs. mutant (-/-) muscle. Each plot represents a separate biological sample. (B) Analysis of RNA sequencing tracks confirmed that Schwann cell-specific genes such as Sox10 and Myelin Protein Zero levels were reduced in erbB3 mutant muscle (-/- #1,-/- #2) to 0.5% and 2.4%, respectively, of values derived from wild-type muscle (+/+ #1, +/+ #2). (C) Functional genomic analysis of genes differentially regulated in diaphragm muscle containing (erbB3 wild-type; +/+) or lacking (erbB3 mutant; -/-) peripheral Schwann cells. (C) Gene Ontology (GO) term networks of the set of genes significantly upregulated in erbB3 mutant vs. WT muscle, overlapped in Cytoscape. The pathways most highly upregulated in erbB3 mutant muscle were related to muscle contractility. In contrast to the upregulation of serpins in muscle from WT mice, there was an increase of serine protease expression in erbB3 mutant muscle. (TIF) [file pgen.1007948.s002.tif]

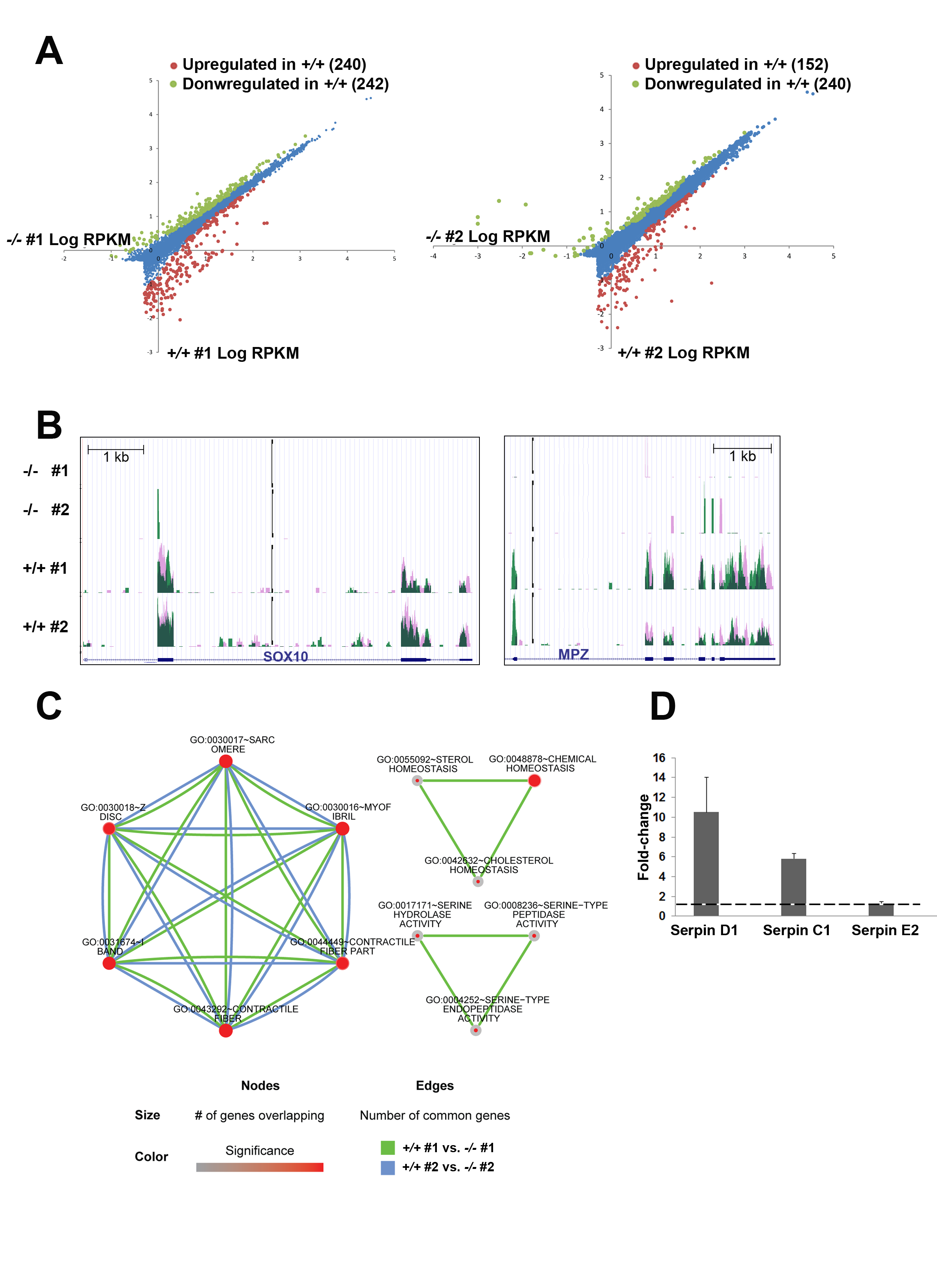

Supplement: S3 Fig — (A) Number of raw and mapped reads in each of two diaphragm samples from erbB3 wild-type (+/+ #1, +/+ #2) vs. mutant (-/- #1, -/- #2) mice at E14.75, as well as comparison of the number of upregulated and downregulated genes between each pair of samples derived from erbB3 wild-type and mutant mice. (B) Gene ontology categories most highly upregulated in erbB3 wild-type sample 1 vs. mutant sample 1 and (C) erbB3 wild-type sample 2 vs. mutant sample 2 show that serine protease inhibitors are highly expressed in wild-type muscle containing Schwann cells vs. erbB3 mutant muscle lacking Schwann cells. (D) qPCR analysis shows that expression of the serpins D1 and C1 are 10-fold and 6-fold higher, respectively, in diaphragm muscle derived from erbB3 wild-type vs. mutant mice at E14.75, whereas expression of serpin E2 is unchanged. Fold-changes are relative to changes in β-actin expression. Dotted line indicates normalized expression of genes in erbB3 mutant muscle. Each value represents (n = 3), samples run in duplicate. (TIF) [file pgen.1007948.s003.tif]

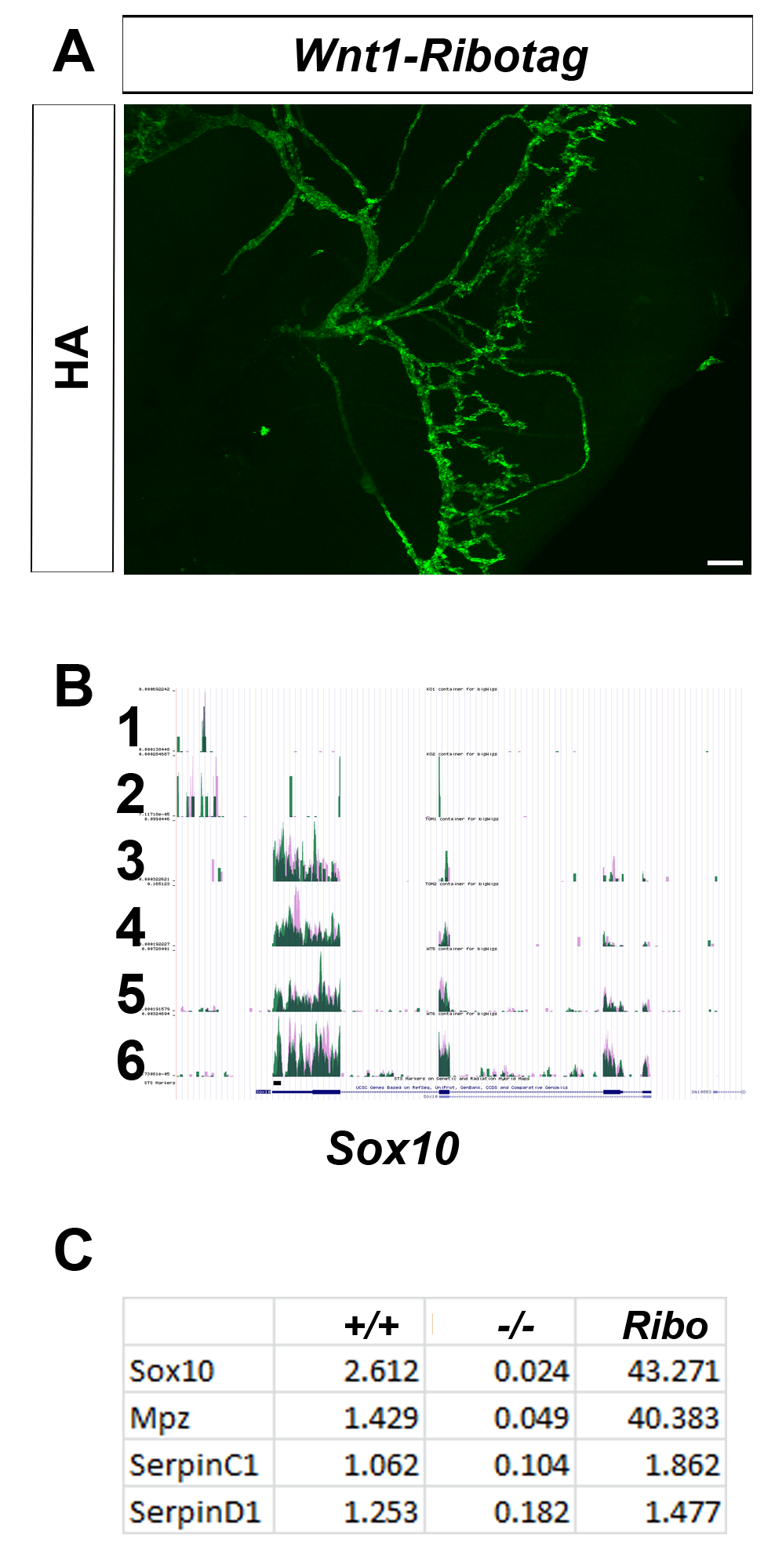

Supplement: S4 Fig — (A) Staining of diaphragm muscle derived from Wnt1-Ribotag (Wnt1-Cre; Rpl22LoxSTOPLox Ribotag) mice at E14.75 with a monoclonal antibody against hemagglutinin (HA) shows robust expression of epitope-tagged ribosomes in Schwann cells along the phrenic nerve. Scale bar = 10 μm. (B) Raw sequencing tracks of Sox10 in diaphragm samples at E14.75 derived from erbB3 mutant mice (Rows 1–2), from WT mice (Rows 3–4), and from Wnt1-Cre, Ribotag mice (Rows 5–6). (C) Reads per kilobase per million mapped read (RPKM) values from muscle-derived samples of the indicated genotypes for the Schwann cell markers Sox10 and myelin protein zero (MPZ) as well as for the anti-thrombins serpinC1 and serpinD1. The enrichment of Sox10 and MPZ in Schwann cells, as determined by Wnt1-Ribotag RPKMs, is higher than for serpinC1 and serpinD1, which may indicate that these proteins are expressed by both muscle and Schwann cells. (TIF) [file pgen.1007948.s004.tif]

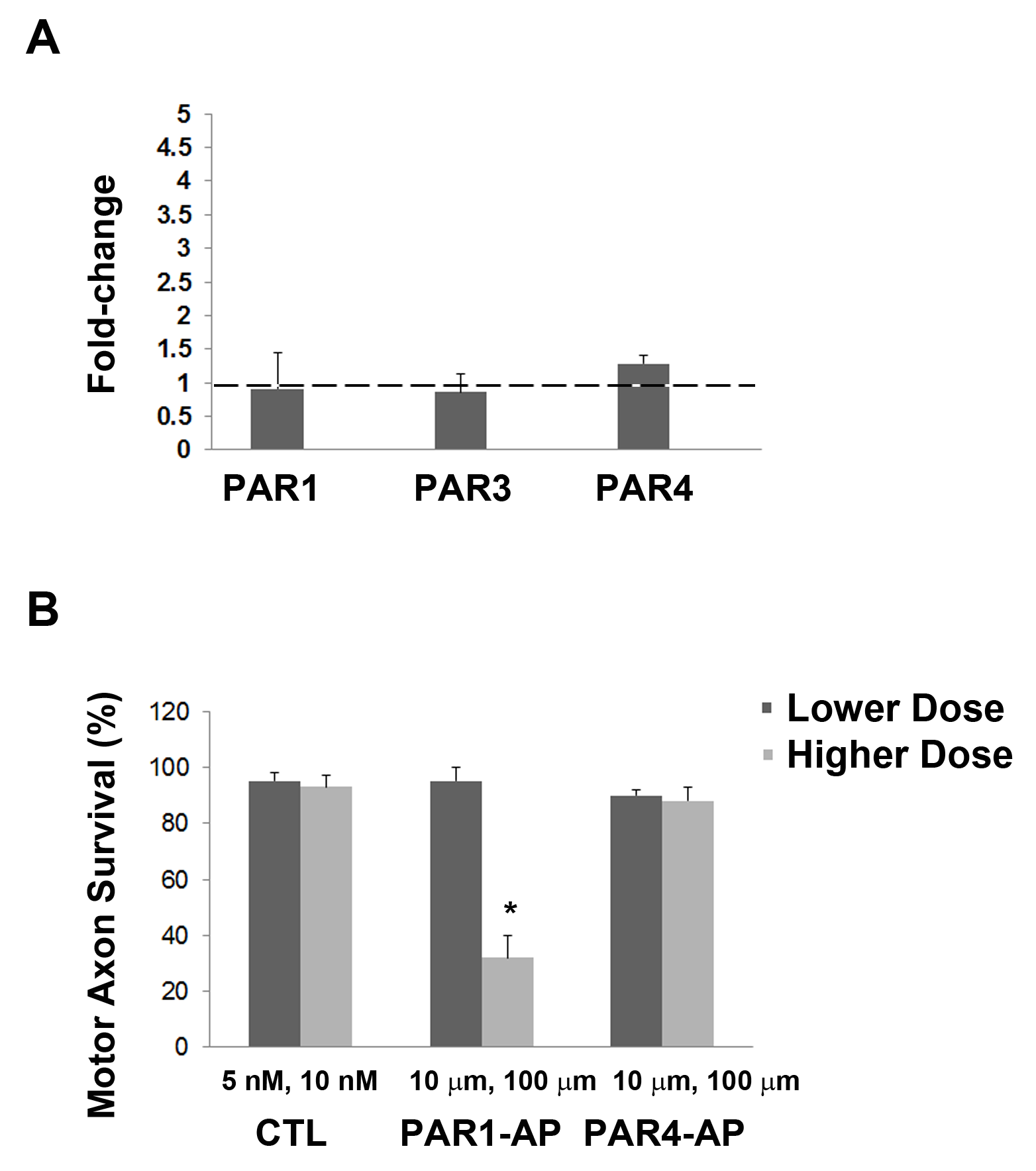

Supplement: S7 Fig — (A) qPCR analysis shows that expression of PAR-1, PAR-3 and PAR-4 is unchanged in the ventral spinal cord at E14.75 of erbB3 wild-type vs. mutant mice. Fold-changes are relative to changes in β-actin expression. Dotted line indicates normalized expression of genes in erbB3 mutant muscle. Each value represents (n = 3), samples run in duplicate. (B) PAR1-AP, at a concentration of 100 μM, but not PAR4-AP, causes significant degeneration of HB9:GFP-positive motor axons when administered 1 day after plating with 5 nM GDNF. CTL refers to 5 nm or 10 nM GDNF treatment at plating and again 1 day after plating. Each value reflects the percentage of healthy motor axons at 2 vs. 1 day after plating, and represents the mean of 3 samples. Dark grey bars = lower dose and light grey bars higher dose of agent. *P<0.01, Student’s t with Bonferroni correction. Scale bar = 200 μm. (TIF) [file pgen.1007948.s007.tif]

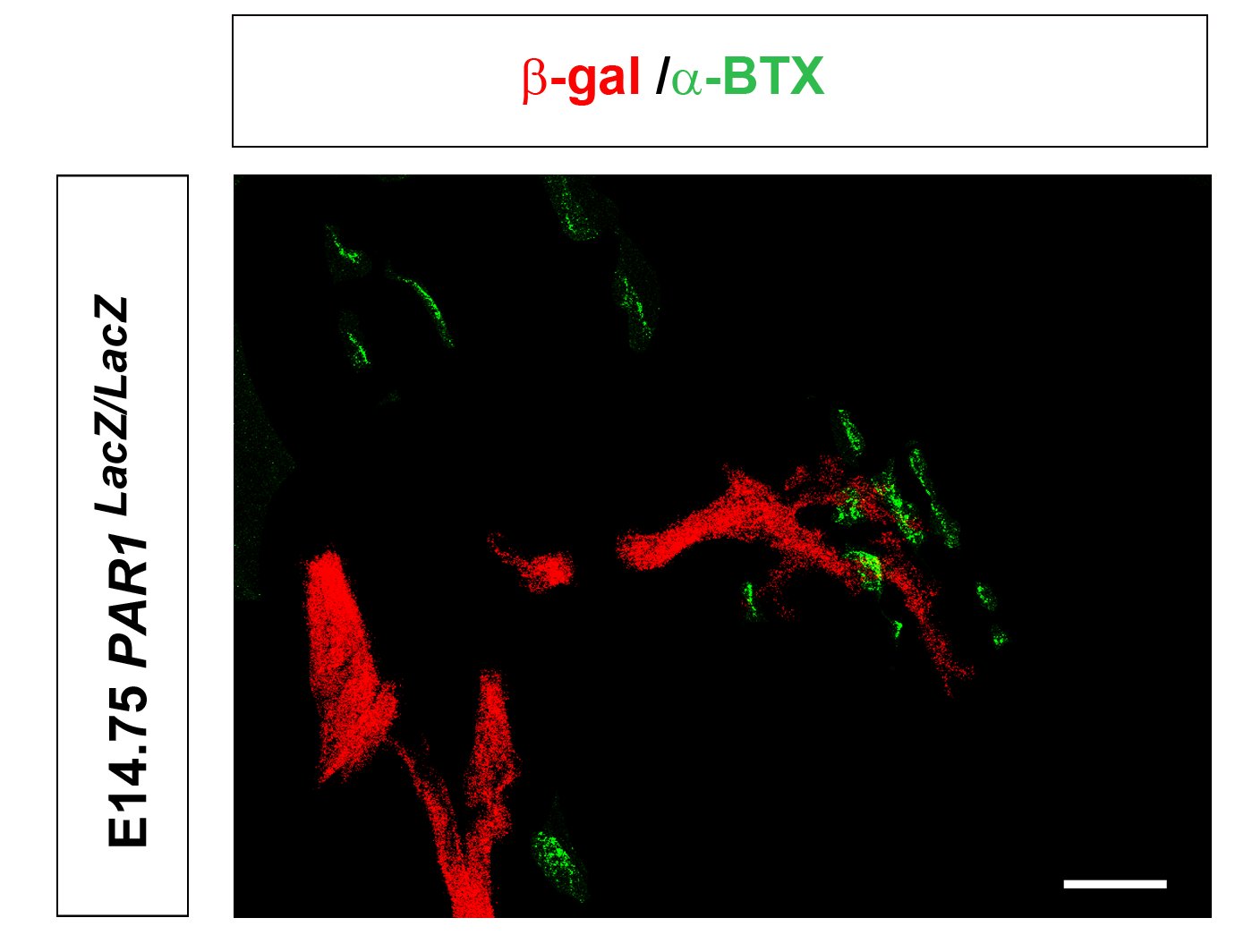

Supplement: S8 Fig — Hindlimbs from PAR1 mutant mice expressing LacZ (PAR1LacZ/LacZ) at E14.75 were sectioned and stained with antibodies against β-galactosidase (β-gal; red) and fluorescent α-BTX (green). Note the staining of motor axons innervating α-BTX-labeled AChRs. Scale bar = 20 μm. (TIF) [file pgen.1007948.s008.tif]
